# New Triterpenoids and Anti-Inflammatory Constituents from *Glinus oppositifolius*

**DOI:** 10.3390/molecules28072903

**Published:** 2023-03-23

**Authors:** Jih-Jung Chen, Chang-Syun Yang, Yu-Hui Chen, Che-Yi Chao, Yu-Chang Chen, Yeuh-Hsiung Kuo

**Affiliations:** 1 Department of Pharmacy, School of Pharmaceutical Sciences, National Yang Ming Chiao Tung University, Taipei 112304, Taiwan; jjungchen@nycu.edu.tw (J.-J.C.); tim0619@nycu.edu.tw (C.-S.Y.); 2 Department of Medical Research, China Medical University Hospital, China Medical University, Taichung 404327, Taiwan; 3 Department of Chinese Pharmaceutical Sciences and Chinese Medicine Resources, China Medical University, Taichung 404327, Taiwan; chenyh2@airproducts.com; 4 Department of Food Nutrition and Health Biotechnology, Asia University, Taichung 41354, Taiwan; chenyj@asia.edu.tw; 5 School of Pharmacy, College of Pharmacy, Kaohsiung Medical University, Kaohsiung 807377, Taiwan; yuchang@kmu.edu.tw; 6 Chinese Medicine Research Center, China Medical University, Taichung 404327, Taiwan; 7 Department of Biotechnology, Asia University, Taichung 413305, Taiwan

**Keywords:** Molluginaceae, *Glinus oppositifolius*, triterpenoids, spergulagenin A, spergulagenin B, spergulagenin C, anti-inflammatory activity

## Abstract

Three new triterpenoids—spergulagenin B (**1**), spergulagenin C (**2**), and spergulagenin D (**3**)—were isolated from the aerial part of *Glinus oppositifolius*, along with 17 known compounds (**4**–**20**). The structures of these new compounds were identified by spectroscopic and MS analyses. Compounds **3**, **5**, **19**, and **20** were evaluated for inhibition of nitric oxide production in LPS-stimulated RAW 264.7 cells with IC_50_ values of 17.03, 18.21, 16.30, and 12.64 μM, respectively. Compounds **3**, **5**, and **20** exhibited inhibitory effects on LPS-induced nitric oxide production in RAW 264.7 cells with IC_50_ values of 18.35 ± 1.34, 17.56 ± 1.41, and 14.27 ± 1.29 μM, respectively.

## 1. Introduction

**Figure 1:**
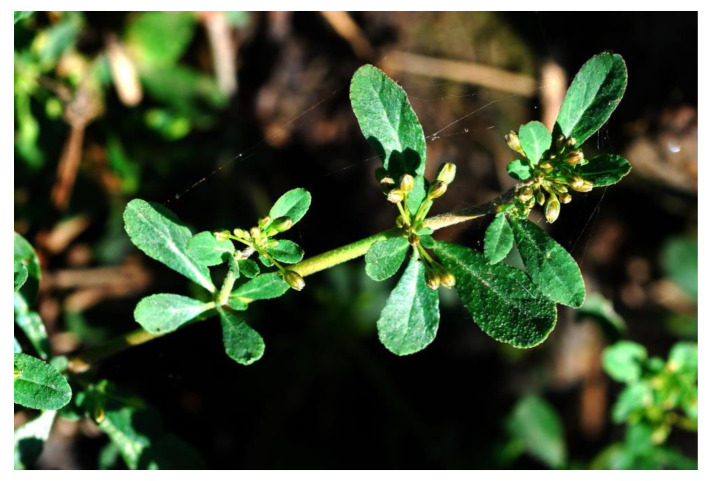
Plant material: *Glinus oppositifolius* (L.) Aug. DC.

Molluginaceae has about 13 genera and more than 120 kinds of plants in the world, mainly distributed in tropical and subtropical regions. *Glinus oppositifolius* (L.) Aug. DC. (Figure 1) is an annual herb mainly distributed at low altitudes in the southern part of Taiwan [1]. *G. oppositifolius* is a folk herb used in the treatment of dermatitis and chronic inflammatory diseases [2]. Flavonoids [3,4], triterpenoids [4], naphthalenes [4], and their derivatives are widely distributed in plants of the family Molluginaceae. Many of these compounds exhibit anti-inflammatory [3,5], antifungal, antiparasitic, and antibacterial activities [6]. Macrophages are one of the immune cells that can secrete nitric oxide (NO), a mediator of inflammatory responses that can participate in host defense [7]. Tumor necrosis factor alpha (TNF-α) is a cytokine with pleiotropic effects on a variety of cell types. It has been recognized as a master regulator of inflammatory responses and has a bearing on the pathogenesis of certain inflammatory diseases [8]. Inhibition of abnormal activation of macrophages by medicines has been proposed as a way to improve inflammatory diseases. *G. oppositifolius* was one of many species that we screened for the anti-inflammatory constituents of Formosan plants. Current phytochemical studies of *G. oppositifoliu* have led to the isolation of three new triterpenoids—spergulagenin B (**1**), spergulagenin C (**2**), and spergulagenin D (**3**)—together with 17 known compounds. This article describes the structural elucidation of **1**–**3** and the anti-inflammatory activity of the isolated compounds.

## 2. Materials and Methods

### 2.1. General

Infrared (IR) spectra (KBr or neat) were measured using a Shimadzu IR prestige-21 Fourier transform infrared spectrophotometer (Shimadzu, Kyoto, Japan). Optical rotations were recorded on a Jasco P-1020 polarimeter (Jasco, Kyoto, Japan) in MeOH and CHCl_3_. Electronic circular dichroism (ECD) spectra were recorded on a Chirascan CD spectrometer (Applied Photophysics Ltd., Leatherhead, UK). High resolution electron ionization mass spectrometry (HR-EI-MS) was measured at Chung Hsing University (Taichung, Taiwan). Ultraviolet (UV) spectra were measured using a Shimadzu Pharmaspec-1700 UV-Visible spectrophotometer (Shimadzu, Kyoto, Japan). Nuclear magnetic resonance (NMR) spectra—including heteronuclear single-quantum coherence (HSQC), correlation spectroscopy (COSY), heteronuclear multiple-bond correlation (HMBC), and nuclear Overhauser effect spectrometry (NOESY) experiments—were measured using a Bruker DRX-500 FT-NMR (Bruker, Bremen, Germany) operating at 125 MHz (^13^C) and 500 MHz (^1^H), respectively. Chemical shifts are given in ppm (δ) using tetramethylsilane (TMS) as internal standard. HPLC separations were carried out utilizing a P230 HPLC system (NATIONAL ANALYTICAL CORPORATION, Maharashtra, India) equipped with P230 HPLC Pump and an IOTA 2 detector, utilizing ChromNav software (version 2.0, Jasco). TLC analysis was performed utilizing aluminum pre-coated Si plates (Merck, Darmstadt, Germany). Column chromatography was carried out utilizing LiChroCART Si gel (5 μM) (Merck, Darmstadt, Germany).

### 2.2. Chemicals

ACS grade solvents (methanol, ethyl acetate, *n*-hexane, acetone, and chloroform), HPLC grade solvents (ethyl acetate, acetone, and *n*-hexane) and deuterated solvents (CDCl_3_, acetone-d_6_, or CD_3_OD) for NMR measurements were procured from Merck, Taipei, Taiwan. LPS (endotoxin from *Escherichia coli*, serotype 0127:B8), Carr (type IV), and quercetin were purchased from MedChemExpress (Monmouth Junction, NJ, USA). MTT (3-[4,5-dimethylthiazol-2-yl]-2,5-diphenyltetrazolium bromide) was acquired from Sigma Chemical Co. (St. Louis, MO, USA).

### 2.3. Plant Material

*Glinus oppositifolius* was collected from Neipu Township, Pingtung County, Taiwan, in February 2010 and identified by J.-J. Chen. A voucher specimen (GO-100514) was deposited in the Department of Pharmacy, National Yang Ming Chiao Tung University, Taipei, Taiwan.

### 2.4. Extraction and Isolation

The dried whole plant (10.58 kg) of *Glinus oppositifolius* was extracted 3 times with methanol (80 L each) for 7 days. The extract was concentrated under reduced pressure at 38 °C, and the residue (1.48 kg) was partitioned between H_2_O and EtOAc (1:1) to provide the EtOAc-soluble fraction (fraction A, 285 g). Fraction A (285 g) was separated by column chromatography (CC) (10.0 kg of SiO_2_, 70–230 mesh; *n*-hexane/EtOAc/methanol gradient) to afford 20 fractions: A1–A20.

Fraction A13 (7.69 g) was purified by Sephadex LH 20 CC (chloroform:methanol = 3:7), silica gel CC (*n*-hexane:acetone = 8:3), and then HPLC (chloroform:acetone = 6:1) to obtain **4** (12.8 mg), **5** (27.4 mg), **6** (12.2 mg), **7** (8.4 mg), and **5** (7.3 mg). Fraction A14 (16.7 g) was purified by silica gel CC (ethyl acetate: *n*-hexane = 1:6) and HPLC (acetone:*n*-hexane = 1:8) to obtain **6** (12.6 mg), **7** (6.4 mg), and **8** (13.4 mg). Fraction A16 (15.5 g) was purified by silica gel CC (*n*-hexane:ethyl acetate = 4:1) and HPLC (*n*-hexane:acetone = 3:1) to obtain **9** (8.2 mg), **10** (27.5 mg), **11** (25.0 mg), **12** (8.4 mg), **13** (13.4 mg), **14** (24.5 mg), **15** (7.8 mg), **16** (6.2 mg), **17** (4.3 mg), **18** (32.4 mg), **19** (4.5 mg), and **20** (32.4 mg). Fraction A18 (13.3 g) was purified by Sephadex LH 20 CC (chloroform:methanol = 3:7), silica gel CC (*n*-hexane:acetone = 5:1), and then semi-preparative HPLC (chloroform: ethyl acetate = 3:2) to obtain **1** (6.6 mg), **2** (4.2 mg), and **3** (3.6 mg).

Spergulagenin B (**1**): colorless needle; mp 306.2–307.6 °C; IR (KBr) υ_max_: 3423 (OH), 2943, 1694 (C=O), 1458, 1385, 1155, 1113, 1061 cm^−1^ (Figure S1); ^1^H-NMR spectroscopic data, see Table 1 (Figure S2); ^13^C-NMR spectroscopic data, see Table 2 (Figure S3); ECD (*c* 0.25, MeOH) *λ*_ma_ (Δ*ε*) 284 (+0.88), 250 (–0.12), 217 (+0.98), 198 (–1.34) nm; HI-EI-MS: 472.3549 [M]^+^ (calcd. for C_30_H_48_O_4_, 472.3547).

Spergulagenin C (**2**): colorless needle; mp 305.4–306.8 °C; UV (MeOH) λ_max_ nm (log λ): 229 (3.73); IR (KBr) υ_max_: 3493 (OH), 3416 (OH), 2972, 1690 (C=O), 1458, 1385, 1355, 1254, 1076 cm^−1^ (Figure S9); ^1^H-NMR spectroscopic data, see Table 1 (Figure S10); ^13^C-NMR spectroscopic data, see Table 2 (Figure S11); ECD (*c* 0.18, MeOH) *λ*_max_ (Δ*ε*) 283 (+0.96), 249 (–0.14), 219 (+1.05), 198 (–1.09) nm; HI-EI-MS: 470.3409 [M]^+^ (calcd. for C_30_H_46_O_4_, 470.3406).

Spergulagenin D (**3**): colorless needle; mp 286.4–287.0 °C; IR (KBr) υ_max_: 3447 (OH), 2938, 1697 (C=O), 1558, 1420, 1387, 1354, 1327 cm^−1^ (Figure S17); ^1^H-NMR spectroscopic data, see Table 1 (Figure S18); ^13^C-NMR spectroscopic data, see Table 2 (Figure S19); ECD (*c* 0.21, MeOH) *λ*_max_ (Δ*ε*) 284 (+1.02), 249 (–0.20), 218 (+1.02), 197 (–0.91) nm; HI-EI-MS: 470.3407 [M]^+^ (calcd. for C_30_H_46_O_4_, 472.3403).

Kaempferol (**4**): yellow powder; mp 274~276 °C; IR (KBr) υ_max_: 3348, 3278~2509, 1661, 1616, 1570, 1089, 1010 cm^−1^; ^1^H -NMR (500 MHz, acetone-d_6_) δ (ppm): 6.26 (1H, d, *J* = 1.9 Hz, H-6), 6.52 (1H, d, *J* = 1.9 Hz, H-8), 7.01 (2H, d, *J* = 8.9 Hz, H-3′ and H-5′), 8.14 (2H, d, *J* = 8.9 Hz, H-2′ and H-6′), 12.15 (1H, s, OH-5).

6,8-Dimethyl-5,7,4′-trihydroxyflavone (**5**): yellow powder; mp 220~225 °C; IR (KBr) υ_max_: 3427, 3704~2509, 1654, 1611, 1576, 1555 cm^−1^; ^1^H-NMR (500 MHz, acetone-d_6_) δ (ppm): 2.13 (3H, s, Me-6), 2.36 (3H, s, Me-8), 6.64 (1H, s, H-3), 7.05 (2H, d, *J* = 8.8 Hz, H-3′, H-5′), 7.98 (2H, d, *J* = 8.8 Hz, H-2′ and H -6′), 13.24 (1H, s, OH-5).

5,7-Dihydroxy-6,8-dimethylflavone (**6**): yellow powder; mp 289~290 °C; IR (KBr) υ_max_: 3400, 3587~2403, 1650, 1602, 1486 cm^−1^; ^1^H-NMR (500 MHz, acetone-d_6_) δ (ppm): 2.19 (3H, s, Me-6), 2.37 (3H, s, Me-8), 6.68 (1H, s, H-3), 7.54 (3H, m, H-3′, H-4′ and H-5′), 7.91 (2H, d, *J* = 7.2 Hz, H-2′ and H-6′), 12.95 (1H, s, OH-5).

5,4′-Dihydroxy-7-methoxy-6,8-dimethylflavone (**7**): yellow powder; mp 286~287 °C; IR (KBr) υ_max_: 3502~2423, 3430, 3072, 2920, 1650, 1612, 1585, 1466 cm^−1^; ^1^H-NMR (500 MHz, CDCl_3_) δ (ppm): 2.18 (3H, s, Me-6), 2.36 (3H, s, Me-8), 3.90 (3H, s, OMe-7), 5.40 (1H, s, OH -4′), 6.89 (1 H, s, H-3), 7.03 (2H, d, *J* = 8.8 Hz, H-3′ and H-5′), 7.87 (2H, d, *J* = 8.8 Hz, H-2′ and H-6′), 13.03 (1H, s, OH-5).

4-Hydroxybenzoic acid (**8**): white solid; mp 210~212 °C; IR (KBr) υ_max_: 3300~2500, 1696 cm^−1^; ^1^H-NMR (500 MHz, CDCl_3_) δ (ppm): 6.33 (1H, br s, Ar-OH), 6.81 (2H, d, *J* = 8.8 Hz, H-3 and H -5), 7.87 (2H, d, *J* = 8.8 Hz, H-2 and H-6).

4-Hydroxybenzaldehyde (**9**): white solid; mp 110~112 °C; IR (KBr) υ_max_: 3170, 1676, 1600, 1519, 1454 cm^−1^; ^1^H-NMR (500 MHz, CDCl_3_) δ (ppm): 5.81 (1H, s, Ar-OH), 6.95 (2H, d, *J* = 8.4 Hz, H-3 and H-5), 7.81 (2H, d, *J* = 8.4 Hz, H-2 and H-6), 9.87 (1H, s, CHO).

4-Hydroxyacetophenone (**10**): white solid; mp 106~107 °C; IR (KBr) υ_max_: 3312, 1664, 1602, 1578 cm^−1^; ^1^H-NMR (500 MHz, CDCl_3_) δ (ppm): 2.56 (3H, s, COMe), 6.09 (1H, s, Ar-OH), 6.89 (2H, d, *J* = 8.8 Hz, H-3 and H-5), 7.91 (2H, d, *J* = 8.8 Hz, H-2 and H-6).

Methyl 4-Hydroxybenzoate (**11**): white solid; mp 124~125 °C; IR (KBr) υ_max_: 3358, 1689, 1608, 1585, 1514 cm^−1^; ^1^H-NMR (500 MHz, CDCl_3_) δ (ppm): 3.89 (3H, s, COOMe), 5.37 (1H, s, Ar-OH), 6.95 (2H, d, *J* = 8.0 Hz, H-3 and H -5), 7.95 (2H, d, *J* = 8.0 Hz, H-2 and H-6).

*p*-Anisic acid (**12**): white solid; mp 182~184°C; IR (KBr) υ_max_: 3307~2503, 2926, 1686, 1605, 1578, 1516 cm^−1^; ^1^H-NMR (500 MHz, CDCl_3_) δ (ppm): 3.85 (3H, s, OMe-4), 6.97 (2H, d, *J* = 8.8 Hz, H-3 and H-5), 7.96 (2H, d, *J* = 8.8 Hz, H-2 and H-6).

Vanillin (**13**): white solid; mp 210~212 °C; IR (KBr) υ_max_: 3213, 2724, 2858, 1667, 1589, 1510 cm^−1^; ^1^H-NMR (500 MHz, CDCl_3_) δ (ppm): 3.97 (3H, s, OMe-3), 6.21 (1H, s, Ar-OH), 7.04 (1H, d, *J* = 8.0 Hz, H-5), 7.42 (1H, d, *J* = 2.0 Hz, H-2), 7.43 (1H, dd, *J* = 8.0, 2.0 and H-6), 9.83 (1H, s, CHO).

4-Hydroxy-3-methoxyacetophenone (**14**): white solid; mp 182~184 °C; IR (KBr) υ_max_: 3323, 2912, 1658, 1575, 1518 cm^−1^; ^1^H-NMR (500 MHz, CDCl_3_) δ (ppm): 2.56 (3H, s, COMe), 3.96 (3H, s, OMe-3), 6.05 (1H, s, Ar-OH), 6.95 (1H, d, *J* = 8.0 Hz), 7.54 (2H, br s, H-2 and H-6).

Acetosyringone (**15**): white solid; mp 105~107 °C; IR (KBr) υ_max_: 3307, 1672, 1608 cm^−1^; ^1^H-NMR (500 MHz, CDCl_3_) δ (ppm): 2.57 (3H, s, COMe), 3.96 (6H, s, OMe-3, OMe-5), 6.05 (1H, s, Ar-OH), 7.25 (2H, s, H-2 and H-6).

4-Hydroxy-3, 5-dimethoxybenzaldehyde (**16**): white solid; mp 112~114 °C; IR (KBr) υ_max_: 3410, 2727, 1685, 1605, 1514 cm^−1^; ^1^H -NMR (500 MHz, CDCl_3_) δ (ppm): 3.98 (6H, s, OMe-3, OMe-5), 5.91 (1H, s, Ar-OH), 7.15 (2H, s, H-2 and H-6), 9.82 (1H, s, CHO).

4-Hydroxybenzyl alcohol (**17**): white solid; mp 116–117 °C; IR (KBr) υ_max_: 3370, 1585, 1512 cm^−1^; ^1^H-NMR (500 MHz, CDCl_3_) δ (ppm): 4.62 (2H, s, H-7), 4.79 (1H, s, Ar-OH), 6.82 (2H, d, *J* = 8.4 Hz, H-3 and H-5), 7.25 (2H, d, *J* = 8.4 Hz, H-2 and H-6).

2-(4-Hydroxyphenyl)ethanol (**18**): white solid; mp 92~93 °C; IR (KBr) υ_max_: 3392, 1599, 1514 cm^−1^; ^1^H-NMR (500 MHz, CDCl_3_) δ (ppm): 2.80 (2H, t, *J* = 8.0 Hz, H-7), 3.83 (2H, br. t, *J* = 8.0 Hz, H-8), 4.75 (1H, s, Ar-OH), 6.79 (2H, d, *J* = 8.0 Hz, H-3 and H-5), 7.10 (2H, d, *J* = 8.0 Hz, H-2 and H-6).

Cinnamic acid (**19**): white solid; mp 133~135 °C; IR (KBr) υ_max_: 3267~2582, 2962, 1684, 1629 cm^−1^; ^1^H-NMR (500 MHz, CDCl_3_) δ (ppm): 6.49 (1H, d, *J* = 16.0 Hz, H-8), 7.41 (3H, m, H-3, H-4 and H-5), 7.60 (2H, dd, *J* = 7.6, 2.0 Hz, H-2 and H-6), 7.68 (1H, d, *J* = 16.0 Hz, H-7).

*trans*-Ferulic acid (**20**): white solid, mp 168~169 °C; IR (KBr) υ_max_: 3435, 3481~2750, 1690, 1662, 1515 cm^−1^; ^1^H-NMR (500 MHz, acetone-d_6_) δ (ppm): 3.89 (3H, s, OMe-3), 6.30 (1H, d, *J* = 15.0 Hz, H-8), 6.80 (1H, d, *J* = 8.0 Hz, H-5), 7.05 (1H, d, *J* = 8.0 Hz, H-6), 7.17 (1H, s, H-2), 7.59 (1H, d, *J* = 15.0 Hz, H-7), 8.17 (1H, br s, Ar-OH).

### 2.5. Cell Culture

Murine RAW264.7 macrophages were cultured in DMEM medium containing 10% fetal bovine serum (FBS) and 1% penicillin at 37 °C, 5% CO_2_ [9].

### 2.6. MTT assay

The MTT assay was performed by the reference method with slight modifications [9].

### 2.7. Nitric Oxide Inhibitory Assay

The NO inhibition assay was followed with a slight modification of the reference method [10].

### 2.8. Enzyme-Linked Immunosorbent Assay

RAW264.7 cells (4 × 10^5^ cells in 96 well plates) were pre-treated with isolated compounds or vehicle (0.05% DMSO) for 1 h and then stimulated with LPS (100 ng/mL) for 20 h. Supernatants were collected and analyzed for production of TNF-α by using appropriate ELISA kits (R&D, Minneapolis, MN, USA) in accordance to the manufacturer’s instructions.

### 2.9. Statistical Analysis

All the data are expressed as mean ± SEM. Statistical analysis was carried out using the Student’s *t*-test. A probability of 0.05 or less was considered statistically significant. All the experiments were performed at least 3 times.

## 3. Results and Discussion

### 3.1. Isolation and Structural Elucidation

**Figure 2:**
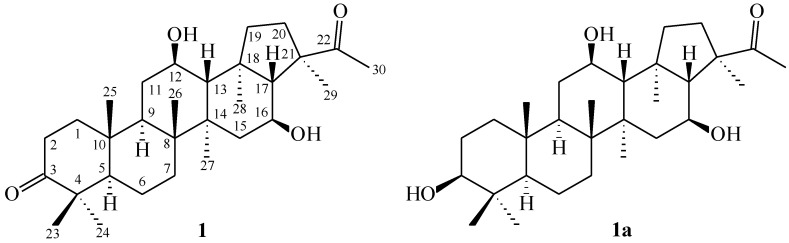

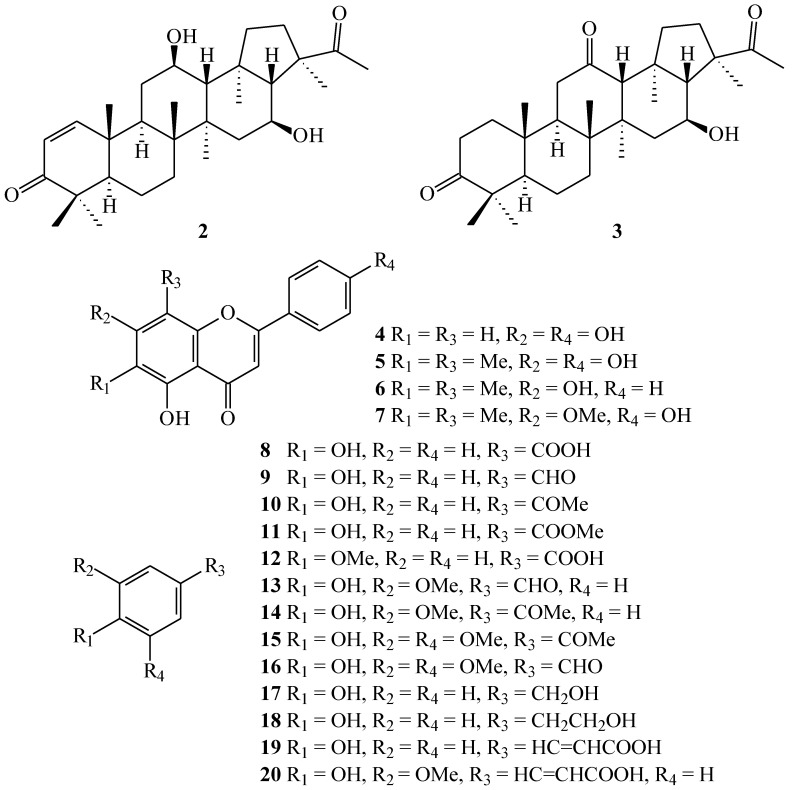
The chemical structures of compounds **1**–**20**.

Chromatographic isolation of the EtOAc-soluble fraction of MeOH extract of aerial part of *G. oppositifolius* on column chromatography and high-performance liquid chromatography (HPLC) afforded three new triterpenoids—spergulagenin B (**1**), spergulagenin C (**2**), and spergulagenin D (**3**)—and 17 known compounds **4**–**20** (Figure 2).

**Figure 3:**
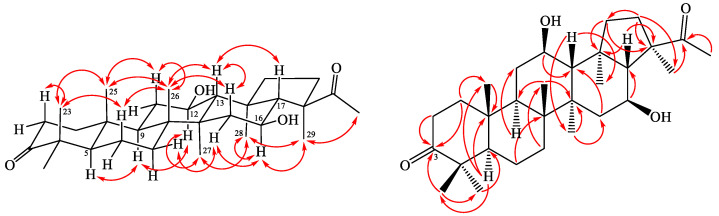
Key NOESY (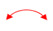) and HMBC (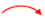) correlations of **1**.

Spergulagenin B (**1**) was isolated as colorless needle with molecular formula C_30_H_48_O_4_ as confirmed by HR-EI-MS, showing an [M]^+^ ion at *m/z* 472.3549 (calcd. 472.3547) and supported by the ^1^H- and ^13^C-NMR data. The IR absorption bands implied the presence of OH (3423 cm^−1^) and acetyl group (1694 cm^−1^). The ^1^H- and ^13^C-NMR data of **1** showed the acetyl group [δ_H_ 2.23 (3H, s, H-30); δ_C_ 25.9 (C-30) and 217.2 (C-22)] and seven methyl signals [δ_H_ 0.96 (3H, s, H-25), 1.01 (3H, s, H-27), 1.03 (3H, s, H-23), 1.04 (3H, s, H-28), 1.07 (3H, s, H-26), 1.08 (3H, s, H-24) and 1.43 (3H, s, H-29); δ_C_ 15.6 (C-25), 16.6 (C-26), 17.2 (C-28), 18.7 (C-27), 21.1 (C-23), 21.2 (C-29) and 26.6 (C-24)]. Comparison of the ^1^H- and ^13^C-NMR data of **1** with those of spergulagenin A (**1a**) [6] suggested that their structures were closely related, except that the carbonyl group [δ_C_ 217.3 (C-3)] at C-3 of **1** replaced the 3β-hydoxyl group of spergulagenin A (**1a**) [6]. This was supported by both HMBC correlations between H-1, H-2, H-23 and C-3 (δ_C_ 217.3). The relative stereochemistry of **1** was elucidated on the basis of NOESY experiments (Figure 2). The NOESY cross-peaks between H-5/H-9, H-9/H-12, H-12/H-27, H-13/H-17, H-13/H-26, H-16/H-29, H-23/H-25, H-25/H-26, H-27/H-28, and, H-28/H-29 suggested that H-13, H-17, Me (23), Me (25) and Me (26) on the β-side and H-5, H-9, H-12, H-16, Me (27), Me (28) and Me (29) are on the α-side of **1**. The full assignment of ^13^C- and ^1^H-NMR resonances was determined by ^13^C-DEPT (Figure S4), ^1^H–^1^H COSY (Figure S5), NOESY (Figure 3 and Figure S6), HSQC (Figure S7), and HMBC (Figure 3 and Figure S8) techniques. The absolute configuration of **1** was evidenced by the ECD Cotton effects at 284 (Δε +0.88), 250 (Δε −0.12), 217 (Δε +0.98), and 198 (Δε −1.34) nm, in analogy with those of glinusopposide D [11]. According to the evidence above, the structure of **1** was elucidated as (3*R*,4*S*,5aR,5b*R*,11a*R*,13*R*,13b*R*)-3-acetyl-4,13-dihydroxy-3,5a,5b,8,8,11a,13b-heptamethylicosahydro-9*H*-cyclopenta[*a*]chrysen-9-one, named spergulagenin B.

**Figure 4:**
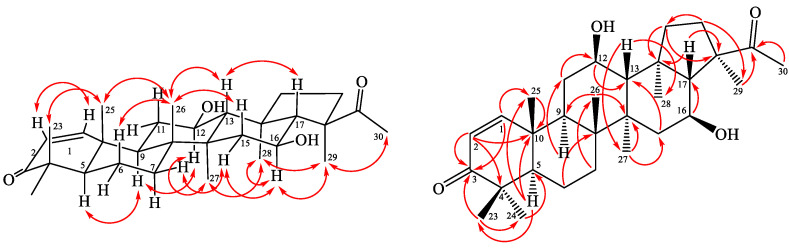
Key NOESY (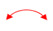) and HMBC (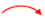) correlations of **1**.

Spergulagenin C (**2**) was obtained as colorless needle crystal. Its molecular formula, C**_30_**H**_46_**O**_4_**, was confirmed by the positive HR-ESI-MS at *m*/*z* 470.3409 [M]^+^ (calculated for C**_30_**H**_46_**O**_4_**, 470.3406) and supported by the ^13^C, ^1^H, and DEPT NMR data. IR absorptions for OH (3493 and 3416 cm^−1^) functions were observed. The presence of the acetyl group was supported by a band at 1690 cm^−1^ in the IR spectrum and was affirmed by signal at δ 25.9, and δ 217.0 in the ^13^C-NMR spectrum. The ^13^C- and ^1^H-NMR data of **2** revealed the acetyl group [δ_H_ 2.24 (3H, s, H-30); δ_C_ 25.9 (C-30) and 217.0 (C-22)] and seven methyl signals [δ_H_ 1.01 (3H, s, H-27), 1.04 (3H, s, H-28), 1.08 (3H, s, H-25), 1.09 (3H, s, H-23), 1.11 (3H, s, H-26), 1.14 (3H, s, H-24), and 1.45 (3H, s, H-29); δ_C_ 17.1 (C-25), 17.2 (C-26), 18.8 (C-28), 18.9 (C-27), 21.4 (C-23), 21.1 (C-29) and 27.8 (C-24)]. The ^1^H- and ^13^C-NMR data of **2** were similar to those of **1**, except that the double bond at C-1,2 [δ_H_ 5.83, 7.10 (each 1H, each d, *J* = 10.0 Hz, H-2 and H-1); δ_C_ 125.6 (C-2), 158.5 (C-1)] of **2** replaced C-1,2 single bond [δ_H_ 1.41, 1.94 (each 1H, m, H-1), 2.42, 2.48 (each 1H, m, H-2); δ_C_ 34.0 (C-2), 39.4 (C-1)] of **1**. This was supported by the HMBC correlations between H-1 (δ_H_ 7.10) and C-3 (δ_C_ 205.3), C-4 (δ_C_ 39.2), C-5 (δ_C_ 53.3), and C-9 (δ_C_ 42.7); and between H-2 (δ_H_ 5.83) and C-4 (δ_C_ 39.2) and C-10 (δ_C_ 44.6). The NOESY cross-peaks between H-5/H-9, H-9/H-12, H-12/H-27, H-13/H-17, H-13/H-26, H-16/H-29, H-23/H-25, H-25/H-26, H-27/H-28, and, H-28/H-29 suggested that H-13, H-17, Me (23), Me (25) and Me (26) are on the β-side and H-5, H-9, H-12, H-16, Me (27), Me (28) and Me (29) are on the α-side of **1**. The full assignment of ^13^C- and ^1^H-NMR resonances was confirmed by ^13^C-DEPT (Figure S12), ^1^H–^1^H COSY (Figure S13), NOESY (Figure 4 and Figure S14), HSQC (Figure S15), and HMBC (Figure 4 and Figure S16) techniques. The absolute configuration of **2** was evidenced by the ECD Cotton effects at 283 (Δε +0.96), 249 (Δε −0.14), 219 (Δε +1.05), and 198 (Δε −1.09) nm, in analogy with those of **1** and glinusopposide D [11]. On the basis of the evidence above, the structure of **2** was elucidated as (3*R*,4*S*,5a*R*,5b*R*,11a*R*,13*R*,13b*R*)-3-acetyl-4,13-dihydroxy-3,5a,5b,8,8,11a,13b-heptamethyl-1,2,3,3a,4,5,5a,5b,6,7,7a,8,11a,11b,12,13,13a,13b-octadecahydro-9*H*-cyclopenta[a] chrysen-9-one, named spergulagenin C.

**Figure 5:**
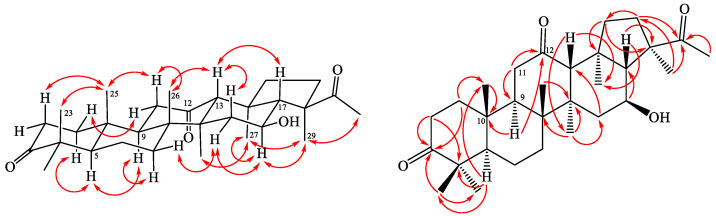
Key NOESY (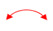) and HMBC (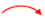) correlations of **1**.

Spergulagenin D (**3**) was obtained as colorless needle. Its molecular formula, C_30_H_46_O_4_, was determined on the basis of the positive HR-EI-MS at *m/z* 470.3407 [M]^+^ (calcd. 470.3403) and supported by the ^1^H, ^13^C, and DEPT NMR data. IR absorptions for OH (3447 cm^−1^) and carbonyl (1697 cm^−1^) functions were observed. The ^1^H- and ^13^C-NMR data of **3** showed the acetyl group [δ_H_ 2.24 (3H, s, H-30); δ_C_ 26.1 (C-30); and 217.3 (C-22)] and seven methyl signals [δ_H_ 0.99 (3H, s, H-27), 1.00 (3H, s, H-25), 1.06 (3H, s, H-23), 1.10 (3H, s, H-24), 1.14 (3H, s, H-28), 1.21 (3H, s, H-26), and 1.43 (3H, s, H-29); δ_C_ 15.2 (C-25), 16.7 (C-26), 17.7 (C-28), 20.9 (C-27), 21.4 (C-23), 21.4 (C-29), and 26.6 (C-24)]. The ^1^H- and ^13^C-NMR data of **3** were similar to those of **1**, except that the carbonyl group at C-12 [δ_C_ 210.9 (C-12)] of **3** replaced the 12β-OH group [δ_H_ 3.95 (each 1H, m, H-12); δ_C_ 69.5 (C-12)] of **1**. This was supported by the HMBC correlations between H-11 (δ_H_ 2.22, 2.25) and C-9 (δ_C_ 49.6), C-12 (δ_C_ 210.9); and between H-9 (δ_H_ 1.70) and C-10 (δ_C_ 37.0) and C-12 (δ_C_ 210.9). The relative stereochemistry of **3** was elucidated on the basis of NOESY experiments (Figure 4). The NOESY cross-peaks between H-5/H-9, H-13/H-17, H-13/H-26, H-16/H-29, H-23/H-25, H-25/H-26, H-27/H-28, and H-28/H-29 suggested that H-13, H-17, Me (23), Me (25) and Me (26) were on the β-side and H-5, H-9, H-16, Me (27), Me (28), and Me (29) were on the α-side of **3**. The full assignment of ^13^C- and ^1^H-NMR resonances was determined by ^13^C-DEPT (Figure S20), ^1^H–^1^H COSY (Figure S21), NOESY (Figure 5 and Figure S22), HSQC (Figure S23), and HMBC (Figure 5 and Figure S24) experiments. The absolute configuration of **3** was evidenced by the ECD Cotton effects at 284 (Δε +0.76), 249 (Δε −0.09), 218 (Δε +1.00), and 197 (Δε −0.91) nm, in analogy with those of **1** and glinusopposide D [11]. On the basis of the evidence above, the structure of **3** was elucidated as (3*R*,4*S*,5a*R*,5b*R*,11a*R*,13b*S*)-3-acetyl-4-hydroxy-3,5a,5b,8,8,11a,13b-heptamethyloctadecahydro-9*H*-cyclopenta[*a*]chrysene-9,13(8*H*)-dione, named spergulagenin D.

### 3.2. Structure Identification of Known Isolated Compounds

The known isolated compounds were readily determined by a comparison of physical and spectroscopic data (^1^H-NMR, ^13^C-NMR, MS, UV, and IR) with the literature values or corresponding authentic samples, and this included four flavonoids, kaempferol (**4**) [12], 6, 8-dimethyl-5, 7, 4′-trihydroxyflavone (**5**) [13], 5,7-dihydroxy-6,8-dimethylflavone (**6**) [14], and 5,4′-dihydroxy-7-methoxy-6,8-dimethylflavone (**7**) [15], and thirteen aromatics, 4-hydroxybenzoic acid (**8**) [16], 4-hydroxybenzaldehyde (**9**) [17], 4-hydroxyacetophenone (**10**) [17], methyl 4-Hydroxybenzoate (**11**) [17], *p*-anisic acid (**12**) [18], vanillin (**13**) [19], 4-hydroxy-3-methoxyacetophenone (**14**) [20], acetosyringone (**15**) [21], 4-hydroxy-3,5-dimethoxybenzaldehyde (**16**) [22], 4-hydroxybenzyl alcohol (**17**) [23], 2-(4-hydroxyphenyl)ethanol (**18**) [24], cinnamic acid (**19**) [25], and trans-ferulic acid (**20**) [26].

### 3.3. Biological Studies

Nitric oxide (NO) is derived from the oxidation of L-arginine by NO synthase (NOS) and is a mediator in the inflammatory response involved in host defense [27]. In inflammation and carcinogenesis conditions, there is an increased production of NO by inducible NO synthase (iNOS) [28]. The anti-inflammatory effects of the compounds isolated from the aerial part of *G. oppositifolius* were also evaluated by suppressing lipopolysaccharide (LPS)-induced NO generation in macrophage cell line RAW264.7. The inhibitory activity data of the isolates **1**–**20** on NO generation by macrophages are shown in Table 3 and Table S1. Quercetin was used as the positive control. From the results of our anti-inflammatory tests, the following conclusions can be drawn: (a) Compounds **3**, **5**, **19**, and **20** exhibited inhibitory effects on lipopolysaccharides (LPS)-induced nitric oxide production in RAW 264.7 cells with IC_50_ values of 17.03 ± 1.28, 18.21 ± 1.15, 16.30 ± 1.41, and 12.64 ± 1.14 μM, respectively (Table 1); (b) Among new triterpenoids, spergulagenin D (**3**) (with 3,12-dioxo groups) exhibited more effective inhibition than its analogues, spergulagenin B (**1**) (with 3-oxo-12β-hydroxy groups) and spergulagenin C (**2**) (with 1,2-dehydro-3-oxo-12β-hydroxy groups) against LPS-induced NO generation. (c) Among the flavonoids, 6,8-dimethyl-5,7,4′-trihydroxyflavone (**5**) (with 6,8-dimethyl-5,7,4′-trihydroxy groups) exhibited more effective inhibition than its analogues, kaempferol (**4**) (with 5,7,4′-trihydroxy groups), 5,7-dihydroxy-6,8-dimethylflavone (**6**) (with 5,7-dihydroxy-6,8-dimethyl groups), and 5,4′-dihydroxy-7-methoxy-6,8-dimethylflavone (**7**) (with 5,4′-dihydroxy-7-methoxy-6,8-dimethyl groups) against LPS-induced NO generation. (d) trans-ferulic acid (**20**) is the most effective among the isolated compounds against LPS-induced NO generation. In addition, compounds **3**, **5**, and **20** exhibited inhibitory effects on LPS-induced TNF-α production in RAW 264.7 cells with IC_50_ values of 18.35 ± 1.34, 17.56 ± 1.41, and 14.27 ± 1.29 μM, respectively (Table 4 and Table S2).

The above findings indicated that the promising inhibitory activity against LPS-induced NO and TNF-α generation of *G. oppositifolius* and its isolates could stimulate future development of new anti-inflammatory agents.

## 4. Conclusions

Twenty compounds, including three new triterpenoids—spergulagenin B (**1**), spergulagenin C (**2**), and spergulagenin D (**3**)—were isolated from aerial part of *G. oppositifolius*. The structures of these new compounds were elucidated on the basis of spectral data. The effects on macrophage pro-inflammatory responses of isolated compounds were evaluated by suppressing LPS-induced NO generation by macrophage RAW264.7 cells. The results of anti-inflammatory assays show that compounds **3**, **5**, **19**, and **20** can obviously inhibit LPS-induced NO generation. Trans-ferulic acid (**20**) is the most effective among the isolated compounds, with IC_50_ value of 12.64 ± 1.14 μM, against LPS-induced NO generation. Furthermore, compounds **3**, **5**, and **20** exhibited inhibitory effects on LPS-induced TNF-α production in RAW 264.7 cells with IC_50_ values of 18.35 ± 1.34, 17.56 ± 1.41, and 14.27 ± 1.29 μM, respectively. Our research indicates *G. oppositifolius* and its isolates (especially **3**, **5**, **19**, and **20**) are worth further research and may be expectantly developed as candidates for the treatment or prevention of various inflammatory diseases (such as dermatitis and arthritis). This study also provides anti-inflammatory scientific evidence for the use of traditional herbal medicine (*G. oppositifolius*) in the treatment of dermatitis and chronic inflammatory diseases [2].

## Figures and Tables

**Table 1:** ^1^H-NMR data for Compounds **1**–**3** (δ in ppm, *J* in Hz).

Position	1 ^a^	2 ^a^	3 ^a^
1	1.94 (m). 1.41 (m)	7.10 (d, *J* = 10.0 Hz)	1.82 (m), 1.39 (m)
2	2.48 (m), 2.42 (m)	5.83 (d, *J* = 10.0 Hz)	2.52 (m), 2.38 (ddd, *J* = 16.0, 5.6, 3.2 Hz)
5	1.30 (m)	1.54 (m)	1.33 (m)
6	1.51 (m), 1.37 (m)	1.56 (m), 1.43 (m)	1.60 (m), 1.35 (m)
7	1.50 (m), 1.31 (m)	1.50 (m), 1.44 (m)	1.54 (m), 1.47 (m)
9	1.69 (m)	1.59 (m)	1.70 (dd, *J* = 9.6, 4.4Hz)
11	1.87 (m), 1.04 (m)	1.57 (m), 1.46 (m)	2.25 (m), 2.22 (m)
12	3.96	3.99 (m)	
13	1.38 (d, *J* = 4.0 Hz)	1.43 (m)	2.23 (m)
15	1.72 (dd, *J* = 12.8, 4.0 Hz), 1.35 (m)	1.72 (m), 1.32 (m)	1.79 (m), 1.44 (m)
16	3.70 (m)	3.71 (m)	3.76 (m)
17	1.76 (d, *J* = 11.2 Hz)	1.78 (m)	1.64 (m)
19	2.02 (m), 1.27 (m)	2.04 (m), 1.28 (m)	2.17 (m), 1.02 (m)
20	2.05 (m), 1.84 (m)	2.05 (m), 1.86 (m)	2.10 (m), 1.93 (m)
23	1.03 (s)	1.09 (s)	1.06 (s)
24	1.08 (s)	1.14 (s)	1.10 (s)
25	0.96 (s)	1.08 (s)	1.00 (s)
26	1.07 (s)	1.11 (s)	1.21 (s)
27	1.01 (s)	1.01 (s)	0.99 (s)
28	1.04 (s)	1.05 (s)	1.14 (2)
29	1.43 (s)	1.45 (s)	1.43 (s)
30	2.23 (s)	2.24 (s)	2.24 (s)

^a^ measured in CDCl_3_ at 500 MHz.

**Table 2:** ^13^C-NMR data for Compounds **1**–**3** (δ in ppm).

Position	1 ^a^	2 ^a^	3 ^a^
1	39.4	158.5	38.9
2	34.0	125.6	34.1
3	217.3	205.3	216.9
4	48.1	39.2	47.6
5	54.9	53.3	55.1
6	19.7	19.1	19.9
7	32.5	32.6	32.0
8	45.5	45.5	47.2
9	47.3	42.7	49.6
10	36.7	44.6	37.0
11	32.9	32.5	39.6
12	69.5	69.3	210.9
13	55.1	55.2	63.4
14	41.4	42.3	41.6
15	45.1	45.0	43.8
16	65.8	65.7	65.8
17	59.2	59.2	58.7
18	46.3	46.3	44.9
19	44.1	44.0	41.7
20	35.9	35.8	35.8
21	54.4	54.4	55.6
22	217.2	217.0	217.3
23	21.1	21.4	21.4
24	26.6	27.8	26.6
25	15.6	17.1	15.2
26	16.6	17.2	16.7
27	18.7	18.9	20.9
28	17.2	18.8	17.7
29	21.2	21.1	21.4
30	25.9	25.9	26.1

^a^ measured in CDCl_3_ at 125 MHz.

**Table 3:** Inhibitory effect of compounds **1**–**20** on production of nitric oxide in LPS-stimulated RAW 264.7 cells.

Compounds	NO Inhibition IC_50_ (μM) ^a^
Spergulagenin B (**1**)	24.76 ± 1.41 ***
Spergulagenin C (**2**)	28.26 ± 2.78 **
Spergulagenin D (**3**)	17.03 ± 1.28
Kaempferol (**4**)	38.87 ± 1.68 ***
6,8-Dimethyl-5,7,4′-trihydroxyflavone (**5**)	18.21 ± 1.15
5,7-Dihydroxy-6,8-dimethylflavone (**6**)	43.61 ± 2.96 ***
5,4′-Dihydroxy-7-methoxy-6,8-dimethylflavone (**7**)	32.08 ± 2.75 **
4-Hydroxybenzoic acid (**8**)	75.83 ± 6.63 **
4-Hydroxybenzaldehyde (**9**)	88.20 ± 7.78 **
4-Hydroxyacetophenone (**10**)	76.24 ± 6.55 **
Methyl 4-Hydroxybenzoate (**11**)	78.50 ± 8.00 **
*p*-Anisic acid (**12**)	115.58 ± 10.35 **
Vanillin (**13**)	94.95 ± 10.99 **
4-Hydroxy-3-methoxyacetophenone (**14**)	111.29 ± 12.91 **
Acetosyringone (**15**)	75.43 ± 6.63 **
4-Hydroxy-3, 5-dimethoxybenzaldehyde (**16**)	86.62 ± 7.74 **
4-Hydroxybenzyl alcohol (**17**)	78.64 ± 7.23 **
2-(4-Hydroxyphenyl)ethanol (**18**)	28.47 ± 1.94 ***
Cinnamic acid (**19**)	16.30 ± 1.41
*trans*-Ferulic acid (**20**)	12.64 ± 1.14 **
Quercetin ^b^	16.74 ± 1.26

^a^ The IC_50_ value was defined as half-maximal inhibitory concentration and was expressed as mean ± SD (n = 3); ^b^ Quercetin was used as positive control; ** *p* < 0.01, and *** *p* < 0.001 compared with the control.

**Table 4:** Inhibitory effect of compounds **1**–**20** on the production of pro-inflammatory cytokine, TNF-α in LPS-stimulated RAW 264.7 cells.

Compounds	TNF-α Inhibition IC_50_ (μM) ^a^
Spergulagenin B (**1**)	30.49 ± 2.20 **
Spergulagenin C (**2**)	31.36 ± 2.59 **
Spergulagenin D (**3**)	18.35 ± 1.34 **
Kaempferol (**4**)	35.71 ± 4.74 *
6,8-Dimethyl-5,7,4′-trihydroxyflavone (**5**)	17.56 ± 1.41 **
5,7-Dihydroxy-6,8-dimethylflavone (**6**)	39.48 ± 3.06 **
5,4′-Dihydroxy-7-methoxy-6,8-dimethylflavone (**7**)	34.17 ± 2.49 **
4-Hydroxybenzoic acid (**8**)	80.02 ± 7.10 **
4-Hydroxybenzaldehyde (**9**)	86.38 ± 6.28 ***
4-Hydroxyacetophenone (**10**)	79.03 ± 5.26 ***
Methyl 4-Hydroxybenzoate (**11**)	82.33 ± 7.25 **
*p*-Anisic acid (**12**)	125.84 ± 11.47 **
Vanillin (**13**)	102.35 ± 9.36 **
4-Hydroxy-3-methoxyacetophenone (**14**)	123.07 ± 11.37 **
Acetosyringone (**15**)	68.38 ± 5.48 **
4-Hydroxy-3, 5-dimethoxybenzaldehyde (**16**)	77.39 ± 6.73 **
4-Hydroxybenzyl alcohol (**17**)	69.38 ± 6.24 **
2-(4-Hydroxyphenyl)ethanol (**18**)	26.44 ± 2.35 *
Cinnamic acid (**19**)	22.00 ± 1.51 **
*trans*-Ferulic acid (**20**)	14.27 ± 1.29 **
Quercetin ^b^	5.08 ± 0.23

^a^ The IC_50_ value was defined as half-maximal inhibitory concentration and was expressed as mean ± SD (n = 3); ^b^ Quercetin was used as positive control; * *p* < 0.05, ** *p* < 0.01, and *** *p* < 0.001 compared with the control.

## Data Availability

The data are contained within the article.

## Supplementary Materials

The following supporting information can be downloaded at: https://www.mdpi.com/article/10.3390/molecules28072903/s1, Figure S1: The IR spectrum of **1**; Figure S2: The ^1^H-NMR spectrum of **1** (CDCl_3_, 500 MHz); Figure S3: The ^13^C-NMR spectrum of **1** (CDCl_3_, 125 MHz); Figure S4: The ^13^C-DEPT spectrum of **1** (CDCl_3_, 125 MHz); Figure S5: The ^1^H–1H COSY spectrum of **1**; Figure S6: The NOESY spectrum of **1**; Figure S7: The HSQC spectrum of **1**; Figure S8: The HMBC spectrum of **1**; Figure S9: The IR spectrum of **2**; Figure S10: The ^1^H-NMR spectrum of **2** (CDCl_3_, 500 MHz); Figure S11: The ^13^C-NMR spectrum of **2** (CDCl_3_, 125 MHz); Figure S12: The ^13^C-DEPT spectrum of **2** (CDCl_3_, 125 MHz); Figure S13: The ^1^H–^1^H COSY spectrum of **2**; Figure S14: The NOESY spectrum of **2**; Figure S15: The HSQC spectrum of **2**; Figure S16: The HMBC spectrum of **2**; Figure S17: The IR spectrum of **3**; Figure S18: The ^1^H-NMR spectrum of **3** (CDCl_3_, 500 MHz); Figure S19: The ^13^C-NMR spectrum of **3** (CDCl_3_, 125 MHz); Figure S20: The ^13^C-DEPT spectrum of **3** (CDCl_3_, 125 MHz); Figure S21: The ^1^H–^1^H COSY spectrum of **3**; Figure S22: The NOESY spectrum of **3**; Figure S23: The HSQC spectrum of **3**; Figure S24: The HMBC spectrum of **3**; Table S1: Inhibitory effect of compounds **1**–**20** on production of nitric oxide in LPS-stimulated RAW 264.7 cells; Table S2: Inhibitory effect of compounds **1**–**20** on the production of pro-inflammatory, TNF-α in LPS-stimulated RAW 264.7 cells.

## Sample Availability

Samples of the compounds are available from the authors.
