# Supplementary material for: New Triterpenoids and Anti-Inflammatory Constituents from Glinus oppositifolius
Source: Molecules. 2023 Mar 23;28(7):2903. doi: 10.3390/molecules28072903 (PMC10096062; doi:10.3390/molecules28072903)
Supplement: Supplementary file 1 [file molecules-28-02903-s001.zip › molecules-2266945-supplementary.pdf]

Supplementary data

New Triterpenoids and Anti-inflammatory Constituents from *Glinus oppositifolius*

Jih-Jung Chen [1,2,†], Chang-Syun Yang [1,†], Yu-Hui Chen [3], Che-Yi Chao [4], Yu-Chang Chen [5] and Yeuh-Hsiung Kuo [3,6,7,*]

[1] Department of Pharmacy, School of Pharmaceutical Sciences, National Yang Ming Chiao Tung University, Taipei 112304, Taiwan; jjungchen@nycu.edu.tw (J.-J.C.); tim0619@nycu.edu.tw (C.-S.Y.)
[2] Department of Medical Research, China Medical University Hospital, China Medical University, Taichung 40402, Taiwan
[3] Department of Chinese Pharmaceutical Sciences and Chinese Medicine Resources, China Medical University, Taichung 40402, Taiwan; chenyh2@airproducts.com (Y.-H.C.)
[4] Department of Food Nutrition and Health Biotechnology, Asia University, Taichung 41354, Taiwan; chenyj@asia.edu.tw
[5] School of Pharmacy, College of Pharmacy, Kaohsiung Medical University, Kaohsiung 807, Taiwan; yuchang@kmu.edu.tw
[6] Chinese Medicine Research Center, China Medical University, Taichung 404, Taiwan
[7] Department of Biotechnology, Asia University, Taichung 413, Taiwan
† These authors contributed equally to this work
* Correspondence: kuoyh@mail.cmu.edu.tw; Tel.: +886-4-2205-3366-5701

Contents

Table S1. Inhibitory effect of compounds **1–20** on production of nitric oxide in LPS-stimulated RAW 264.7 cells... S4

Table S2. Inhibitory effect of compounds **1–20** on the production of pro-inflammatory cytokine, TNF-α in LPS-stimulated RAW 264.7 cells.. S5

Figure S1. The IR spectrum of **1**.. S6

Figure S2. The ^{1}H-NMR spectrum of **1** ($CDCl_3$, 500 MHz)... S6

Figure S3. The ^{13}C-NMR spectrum of **1** ($CDCl_3$, 125 MHz)... S7

Figure S4. The ^{13}C-DEPT spectrum of **1** ($CDCl_3$, 125 MHz)... S7

Figure S5. The ^{1}H–^{1}H COSY spectrum of **1**.. S8

Figure S6. The NOESY spectrum of **1**.. S8

Figure S7. The HSQC spectrum of **1**.. S9

Figure S8. The HMBC spectrum of **1**.. S9

Figure S9. The IR spectrum of **2**... S10

Figure S10. The ^{1}H-NMR spectrum of **2** ($CDCl_3$, 500 MHz).. S10

Figure S11. The ^{13}C-NMR spectrum of **2** ($CDCl_3$, 125 MHz).. S11

Figure S12. The ^{13}C-DEPT spectrum of **2** ($CDCl_3$, 125 MHz).. S11

Figure S13. The ^{1}H–^{1}H COSY spectrum of **2**... S12

Figure S14. The NOESY spectrum of **2**... S12

Figure S15. The HSQC spectrum of **2**... S13

Figure S16. The HMBC spectrum of **2**... S13

Figure S17. The IR spectrum of **3**... S14

Figure S18. The ^{1}H-NMR spectrum of **3** ($CDCl_3$, 500 MHz).. S14

Figure S19. The ^{13}C-NMR spectrum of **3** ($CDCl_3$, 125 MHz)..S15

Figure S20. The ^{13}C-DEPT spectrum of **3** ($CDCl_3$, 125 MHz).. S15

Figure S21. The ^{1}H–^{1}H COSY spectrum of **3**... S16

Figure S22. The NOESY spectrum of **3**... S16

Figure S23. The HSQC spectrum of **3**...S17

Figure S24. The HMBC spectrum of **3**..S17

Table S1. Inhibitory effect of compounds **1–20** on production of nitric oxide in LPS-stimulated RAW 264.7 cells.

**Compounds**	**N1**	**N2**	**N3**	**NO inhibition IC_{50} (μM)[a]**
Spergulagenin B (**1**)	25.00	23.35	25.93	24.76 ± 1.41 ***
Spergulagenin C (**2**)	31.04	25.59	28.15	28.26 ± 2.78 **
Spergulagenin D (**3**)	15.79	16.99	18.31	17.03 ± 1.28
Kaempferol (**4**)	40.55	39.01	37.33	38.87 ± 1.68 ***
6,8-Dimethyl-5,7,4′-tri-hydroxyflavone (**5**)	18.33	19.24	17.06	18.21 ± 1.15
5,7-Dihydroxy-6,8-dimethylflavone (**6**)	43.82	46.46	40.65	43.61 ± 2.96 ***
5,4′-Dihydroxy-7-methoxy-6,8-dimethylflavone (**7**)	31.95	29.46	34.83	32.08 ± 2.75 **
4-Hydroxybenzoic acid (**8**)	69.20	76.00	82.29	75.83 ± 6.63 **
4-Hydroxybenzaldehyde (**9**)	95.98	80.63	87.99	88.20 ± 7.78 **
4-Hydroxyacetophenone (**10**)	69.90	76.03	82.79	76.24 ± 6.55 **
Methyl 4-Hydroxybenzoate (**11**)	78.44	70.56	86.50	78.50 ± 8.00 **
p-Anisic acid (**12**)	125.81	105.23	115.70	115.58 ± 10.35 **
Vanillin (**13**)	83.96	105.88	95.01	94.95 ± 10.99 **
4-Hydroxy-3-methoxy-acetophenone (**14**)	123.20	111.02	98.65	111.29 ± 12.91 **
Acetosyringone (**15**)	75.37	68.86	82.06	75.43 ± 6.63 **
4-Hydroxy-3, 5-dimethoxy-benzaldehyde (**16**)	86.50	94.36	79.00	86.62 ± 7.74 **
4-Hydroxybenzyl alcohol (**17**)	78.72	85.79	71.41	78.64 ± 7.23 **
2-(4-Hydroxyphenyl)ethanol (**18**)	30.33	28.55	26.53	28.47 ± 1.94 ***
Cinnamic acid (**19**)	15.20	17.71	15.99	16.30 ± 1.41
trans-Ferulic acid (**20**)	13.87	12.51	11.63	12.64 ± 1.14 **
Quercetin [b]	17.94	16.80	15.48	16.74 ± 1.26

[a] The IC_{50} value was defined as half-maximal inhibitory concentration and was expressed as mean ± SD (n = 3); [b] Quercetin was used as positive control; * $p < 0.05$, ** $p < 0.01$, *** $p < 0.001$ compared with the control.

Table S2. Inhibitory effect of compounds **1–20** on the production of pro-inflammatory cytokine, TNF-*α* in LPS-stimulated RAW 264.7 cells.

**Compounds**	**N1**	**N2**	**N3**	**TNF-*α* inhibition IC_{50} (μM)[a]**
Spergulagenin B (**1**)	28.54	30.24	32.69	30.49 ± 2.20 **
Spergulagenin C (**2**)	33.95	31.00	29.13	31.36 ± 2.59 **
Spergulagenin D (**3**)	18.24	19.69	17.12	18.35 ± 1.34 **
Kaempferol (**4**)	30.97	40.71	35.45	35.71 ± 4.74 *
6,8-Dimethyl-5,7,4′-tri-hydroxyflavone (**5**)	16.15	19.30	17.23	17.56 ± 1.41 **
5,7-Dihydroxy-6,8-dimethylflavone (**6**)	39.21	42.81	36.42	9.48 ± 3.06 **
5,4′-Dihydroxy-7-methoxy-6,8-dimethylflavone (**7**)	31.68	36.27	34.56	34.17 ± 2.49 **
4-Hydroxybenzoic acid (**8**)	72.97	87.12	79.97	80.02 ± 7.10 **
4-Hydroxybenzaldehyde (**9**)	80.49	92.66	85.99	86.38 ± 6.28 ***
4-Hydroxyacetophenone (**10**)	84.29	78.97	73.83	79.03 ± 5.26 ***
Methyl 4-Hydroxybenzoate (**11**)	75.40	82.01	89.58	82.33 ± 7.25 **
p-Anisic acid (**12**)	137.14	126.01	114.37	125.84 ± 11.47 **
Vanillin (**13**)	111.71	102.22	93.12	102.35 ± 9.36 **
4-Hydroxy-3-methoxy-acetophenone (**14**)	122.90	111.87	134.44	123.07 ± 11.37 **
Acetosyringone (**15**)	73.86	68.17	63.11	68.38 ± 5.48 **
4-Hydroxy-3, 5-dimethoxy-benzaldehyde (**16**)	70.80	77.25	84.12	77.39 ± 6.73 **
4-Hydroxybenzyl alcohol (**17**)	69.50	61.62	77.02	69.38 ± 7.64 **
2-(4-Hydroxyphenyl)ethanol (**18**)	24.14	28.79	26.39	26.44 ± 2.35 *
Cinnamic acid (**19**)	20.54	23.51	21.95	22.00 ± 1.51 **
trans-Ferulic acid (**20**)	14.38	15.45	14.27	14.27 ± 1.29 **
Quercetin [b]	5.28	5.11	4.85	5.08 ± 0.23

[a] The IC_{50} value was defined as half-maximal inhibitory concentration and was expressed as mean ± SD (n = 3); [b] Quercetin was used as positive control; * $p < 0.05$, **$p < 0.01$, and ***$p < 0.001$ compared with the control.

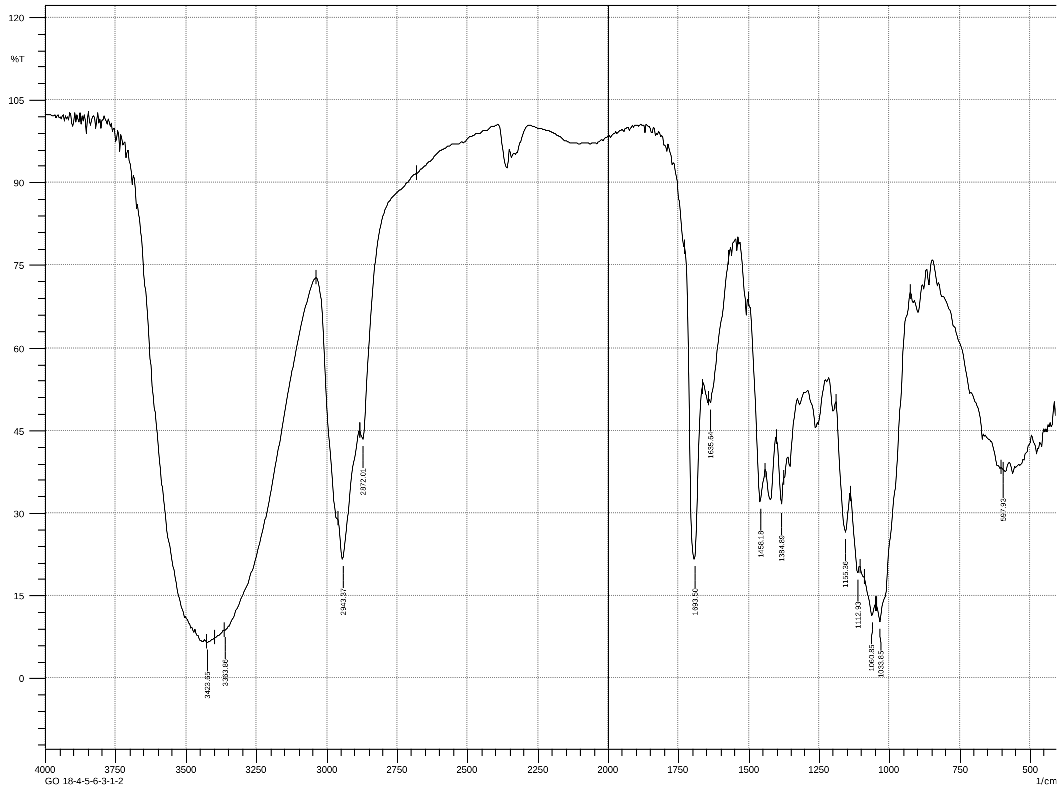

Figure S1. IR spectrum of **1**.

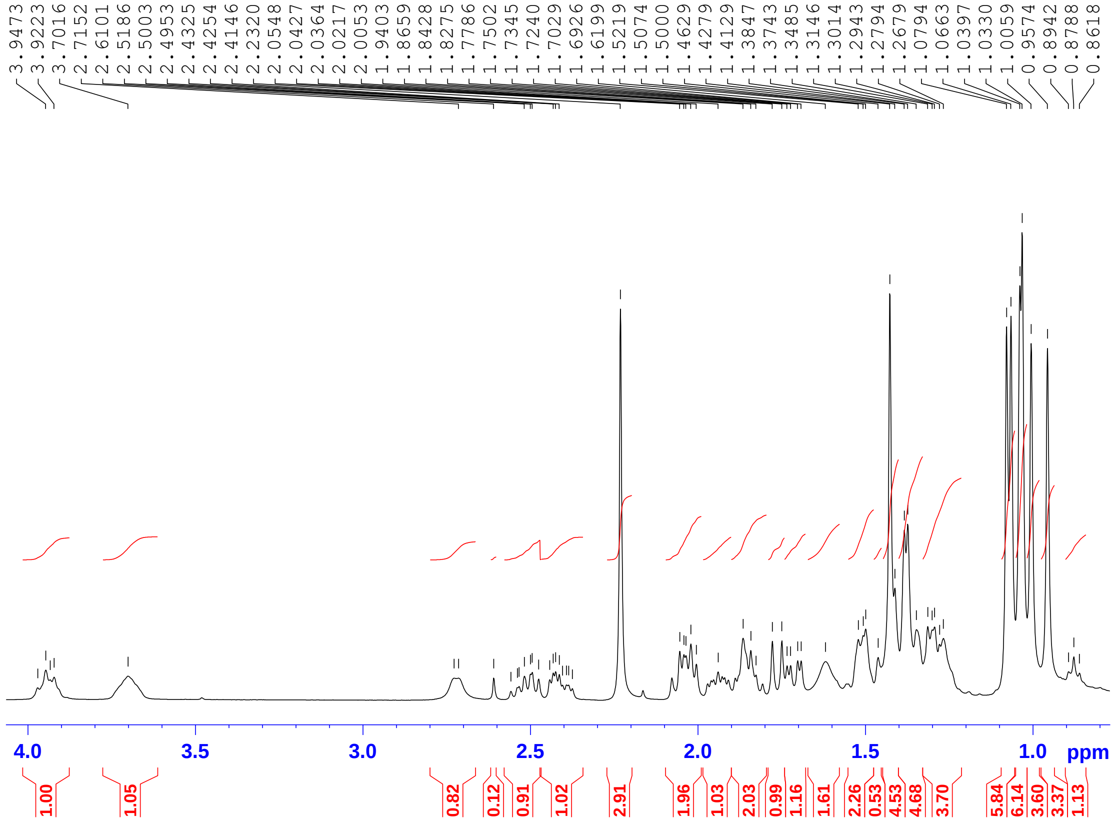

Figure S2. ^{1}H-NMR spectrum of **1** ($CDCl_3$, 500 MHz).

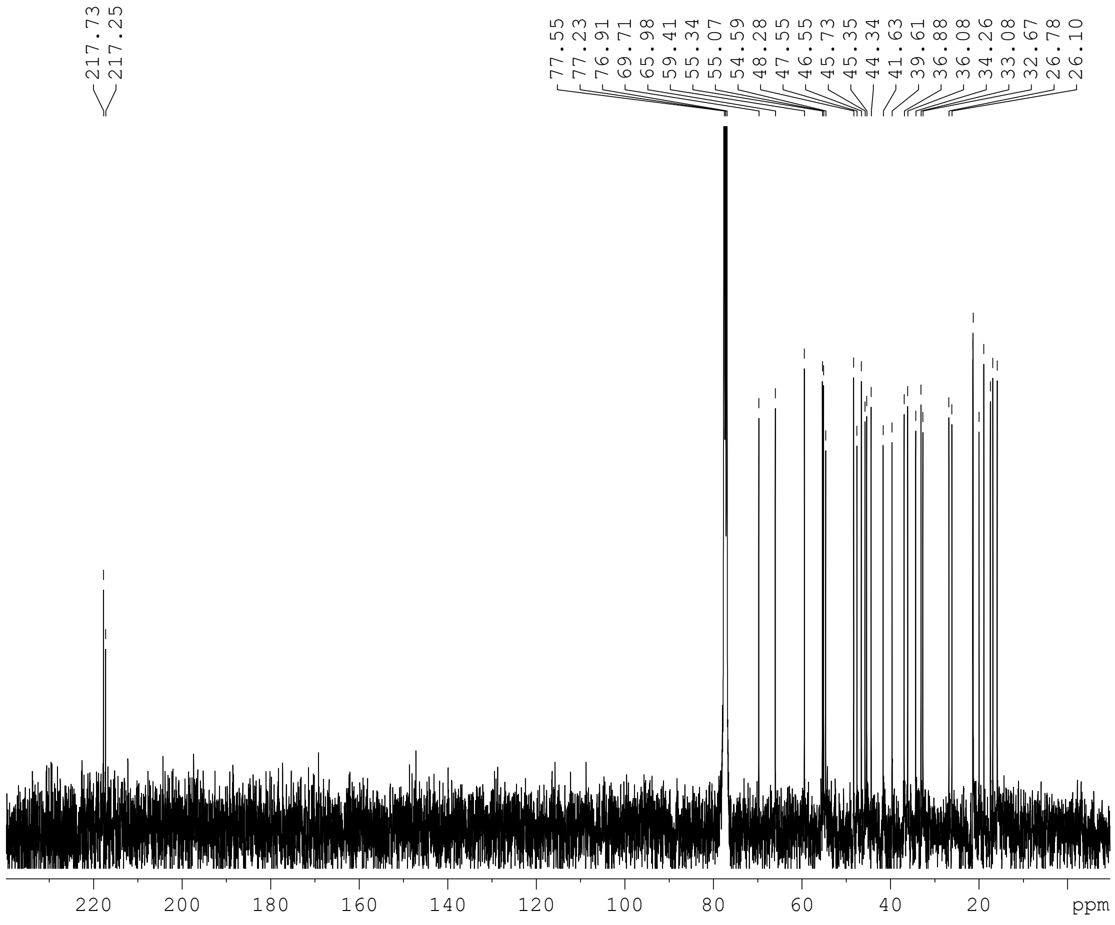

Figure S3. ^{13}C-NMR spectrum of **1** ($CDCl_3$, 125 MHz).

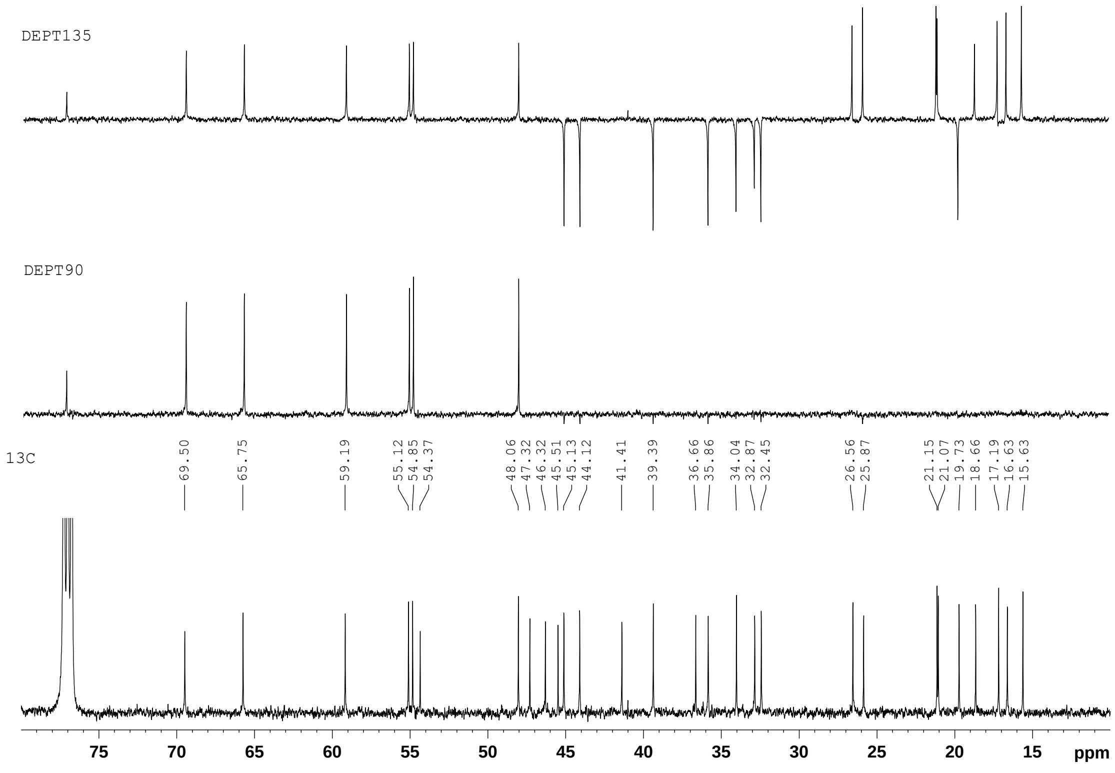

Figure S4. ^{13}C-DEPT spectrum of **1** ($CDCl_3$, 125 MHz).

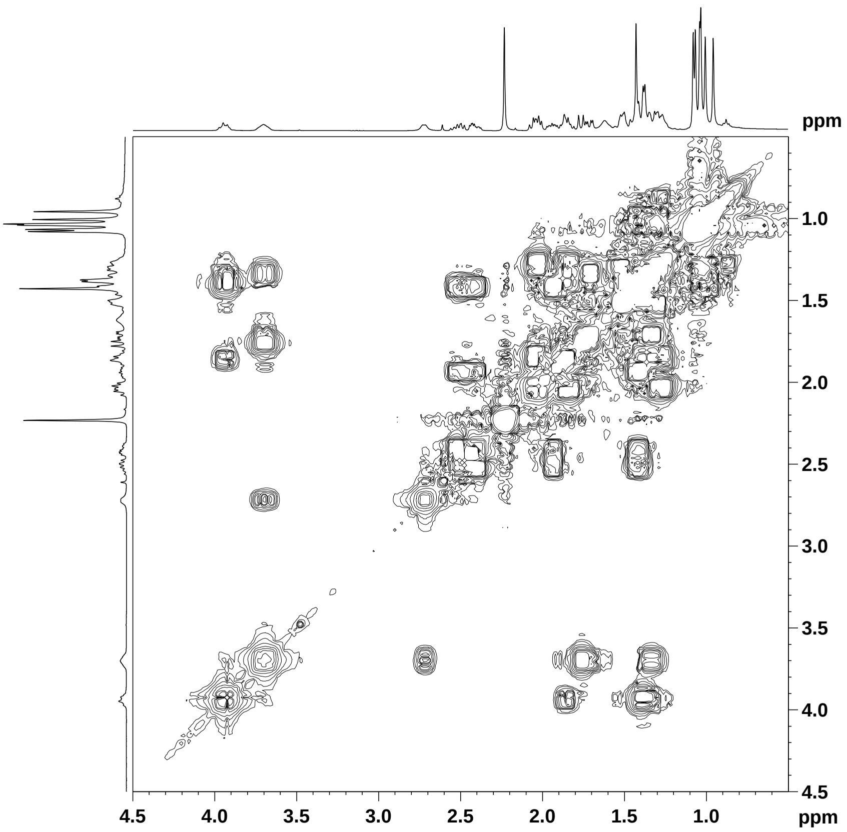

Figure S5.^{1}H–^{1}H COSY spectrum of **1**.

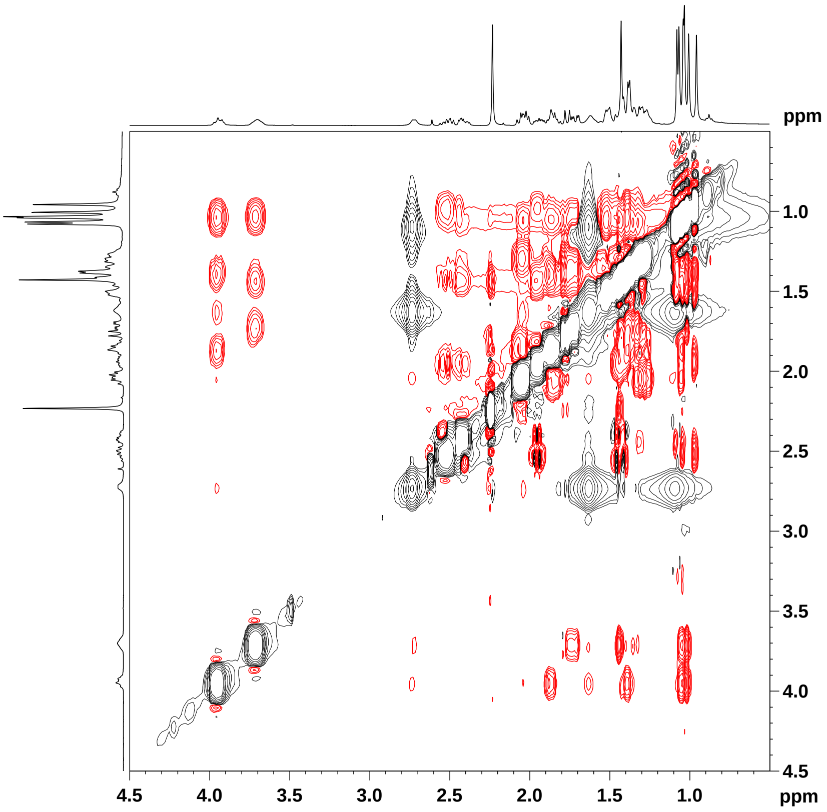

Figure S6. NOESY spectrum of **1**.

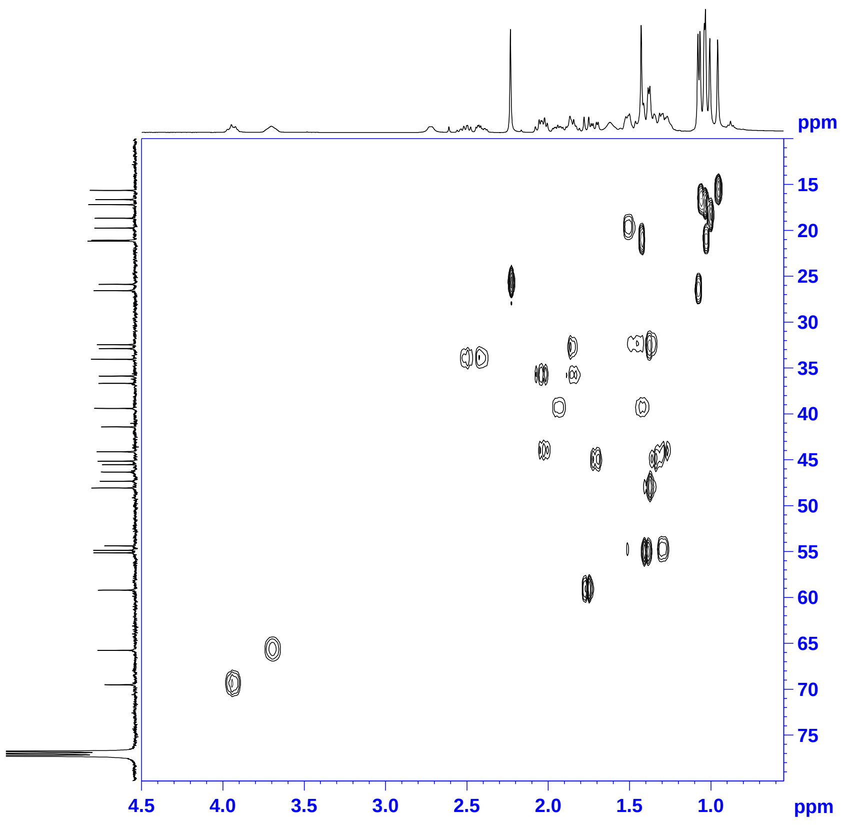

Figure S7. HSQC spectrum of **1**.

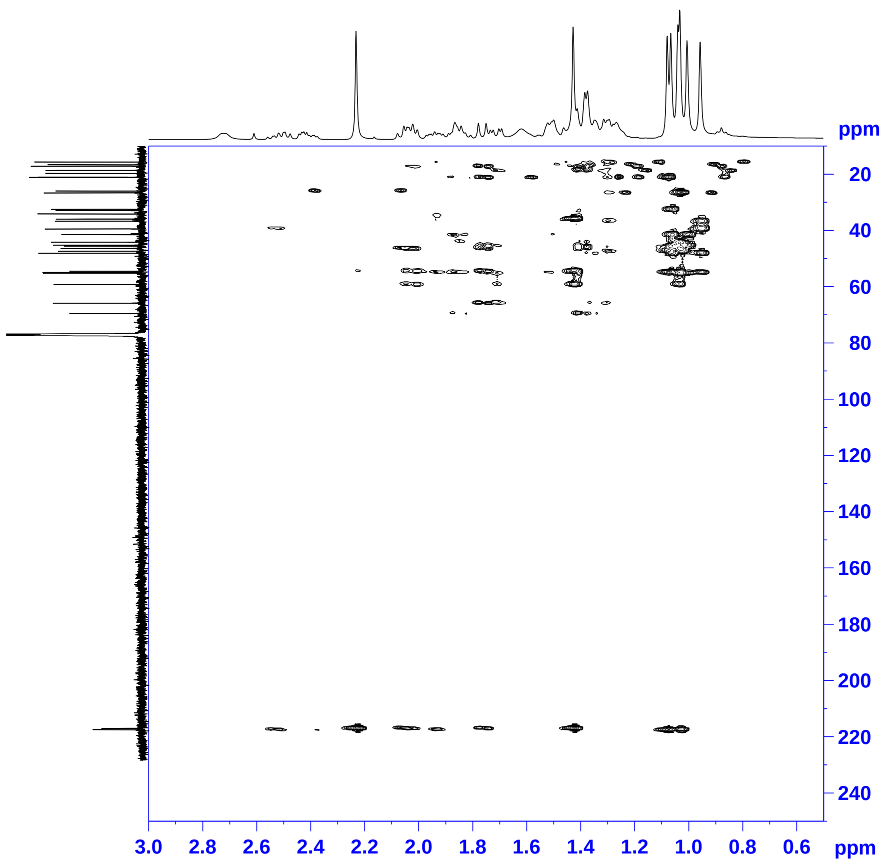

Figure S8. HMBC spectrum of **1**.

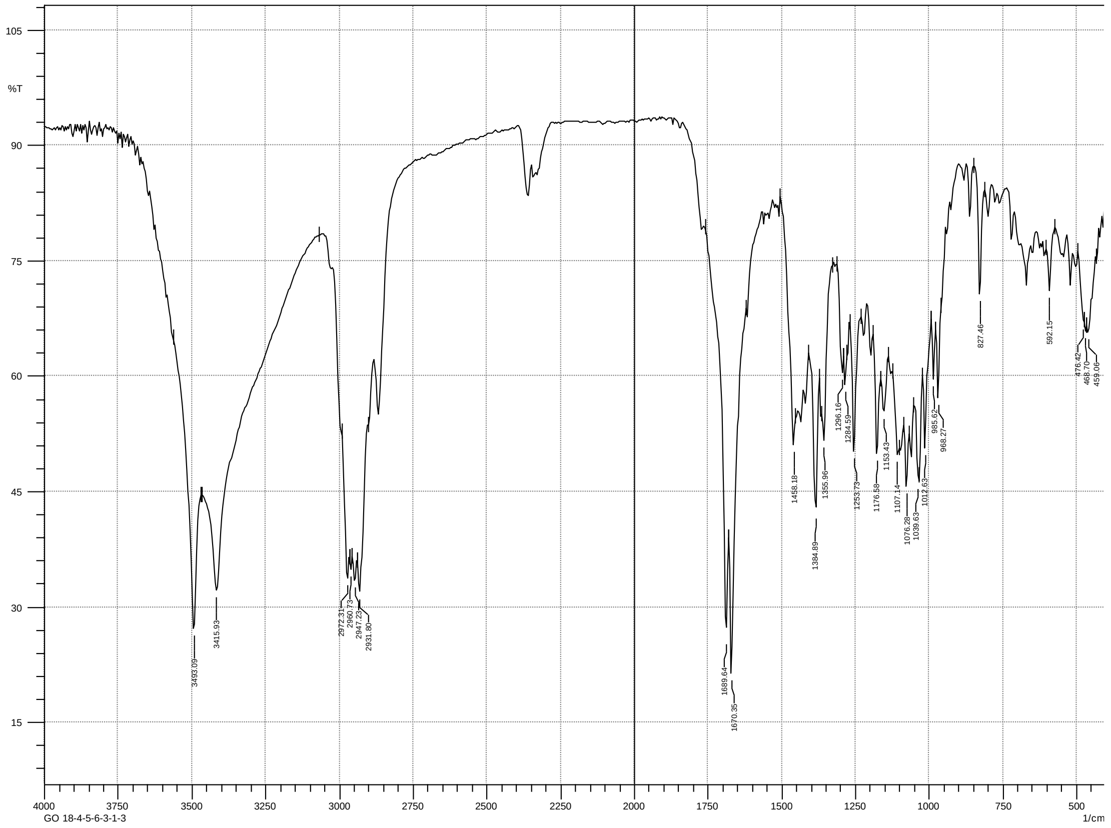

Figure S9. IR spectrum of **2.**

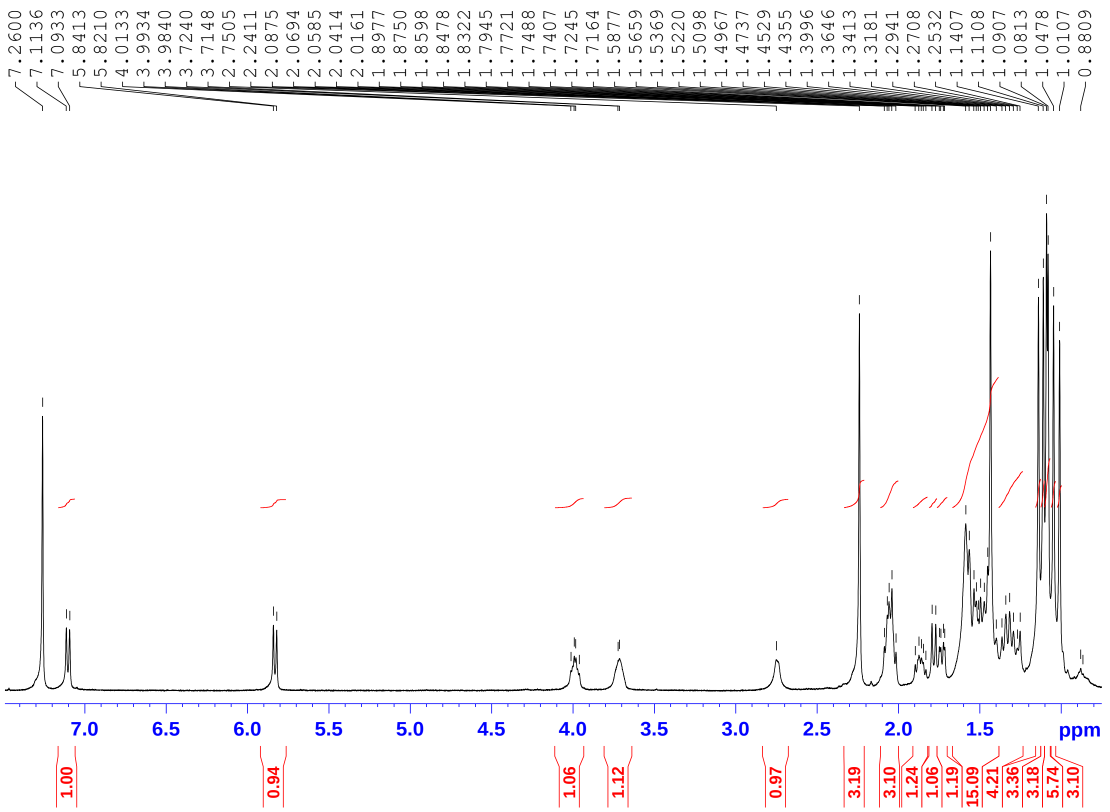

Figure S10. ^{1}H-NMR spectrum of **2** ($CDCl_3$, 500 MHz).

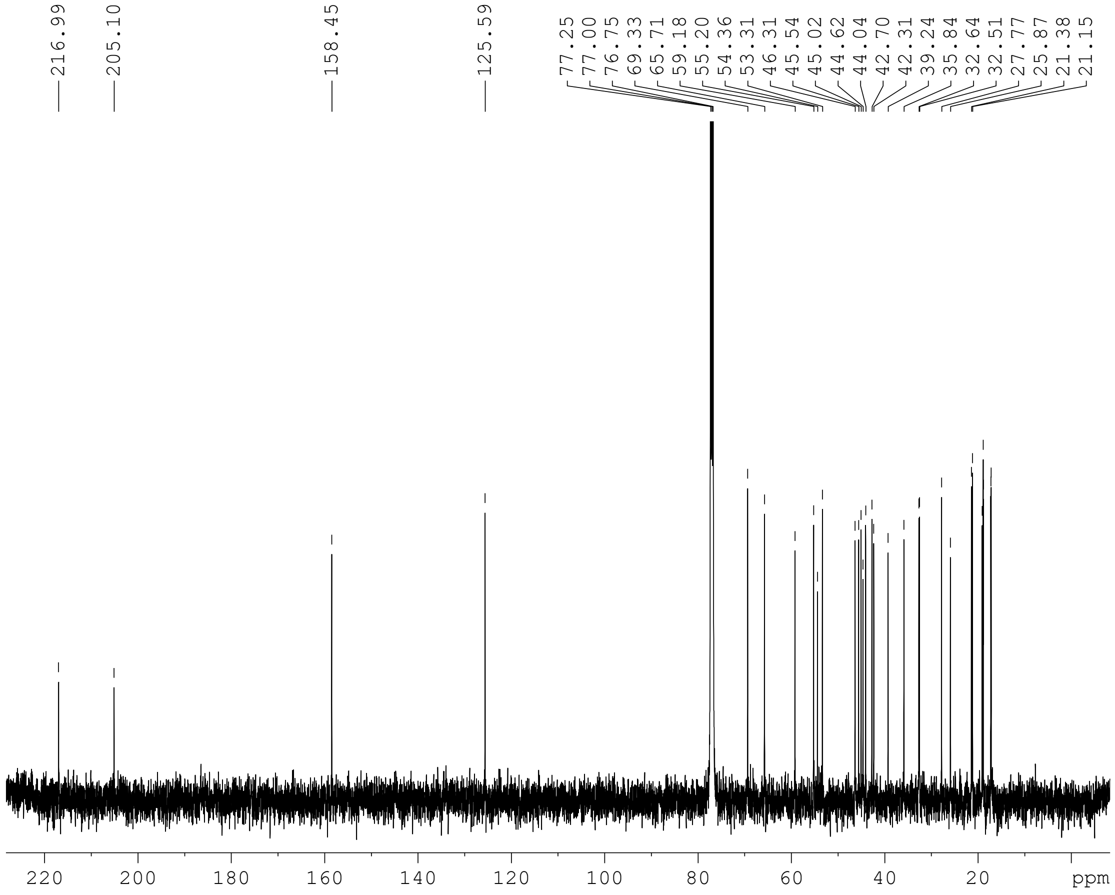

Figure S11.^{13}C-NMR spectrum of **2** ($CDCl_3$, 125 MHz).

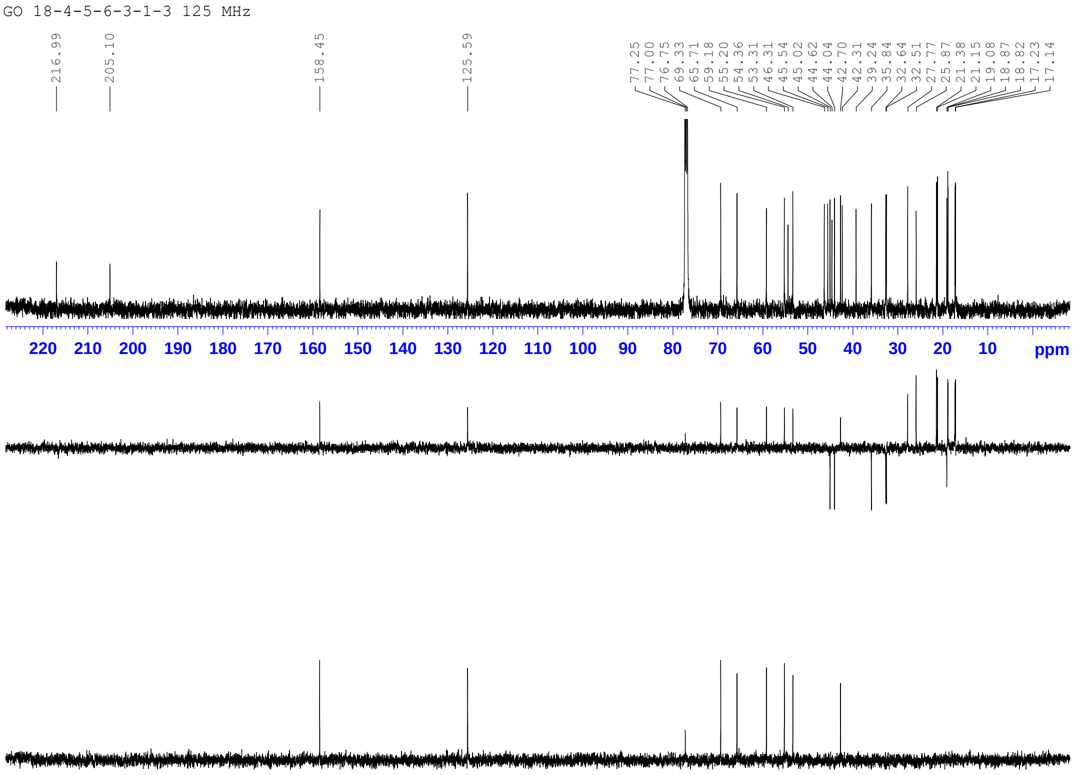

Figure S12. ^{13}C-DEPT spectrum of **2** ($CDCl_3$, 125 MHz).

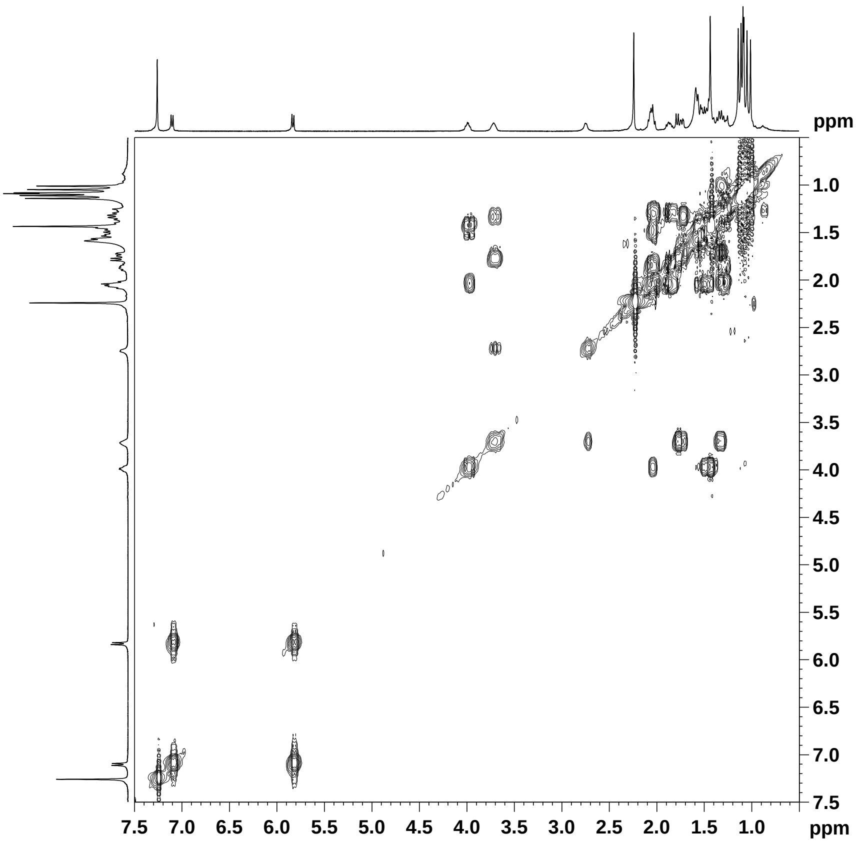

Figure S13. ^{1}H–^{1}H COSY spectrum of **2**.

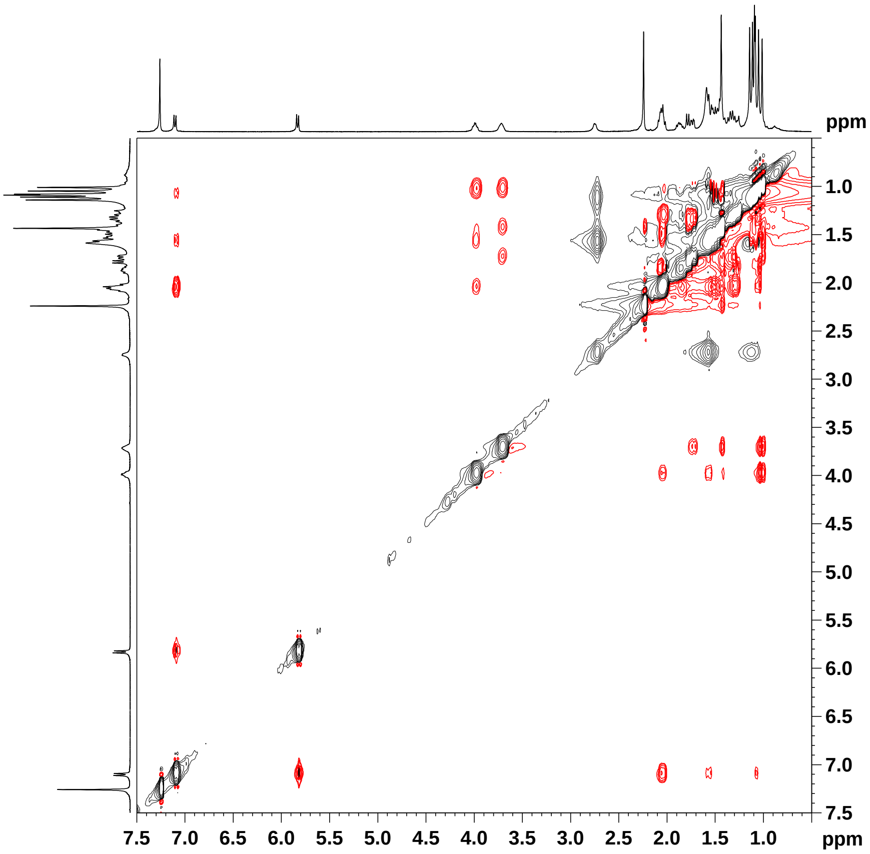

Figure S14. NOESY spectrum of **2**.

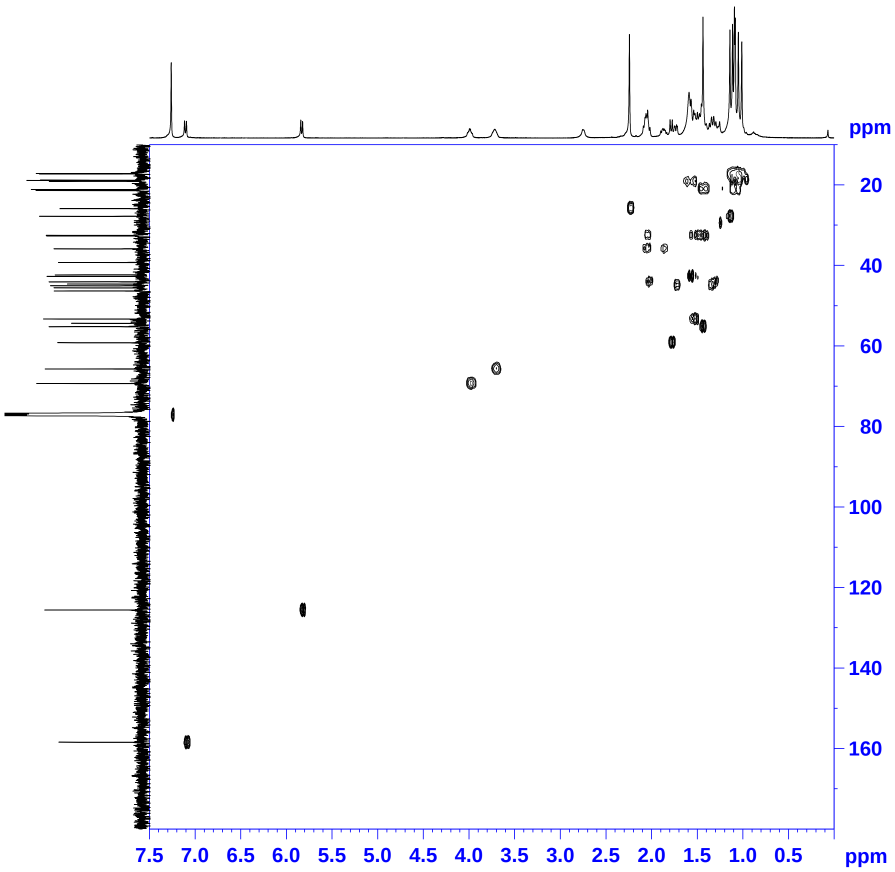

Figure S15.HSQC spectrum of **2**.

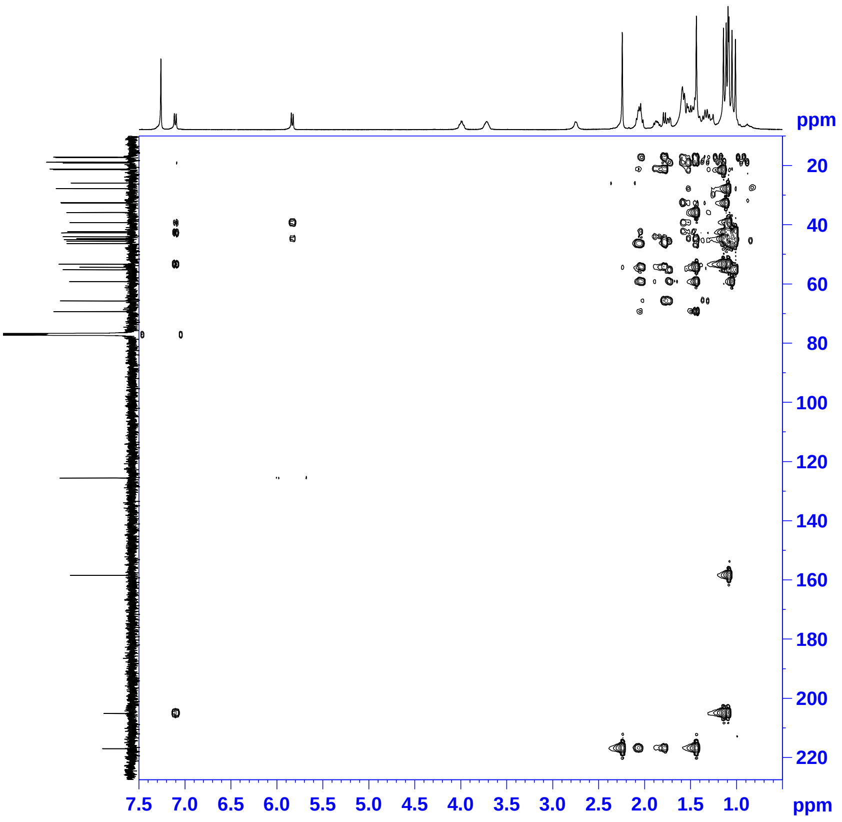

Figure S16. HMBC spectrum of **2**.

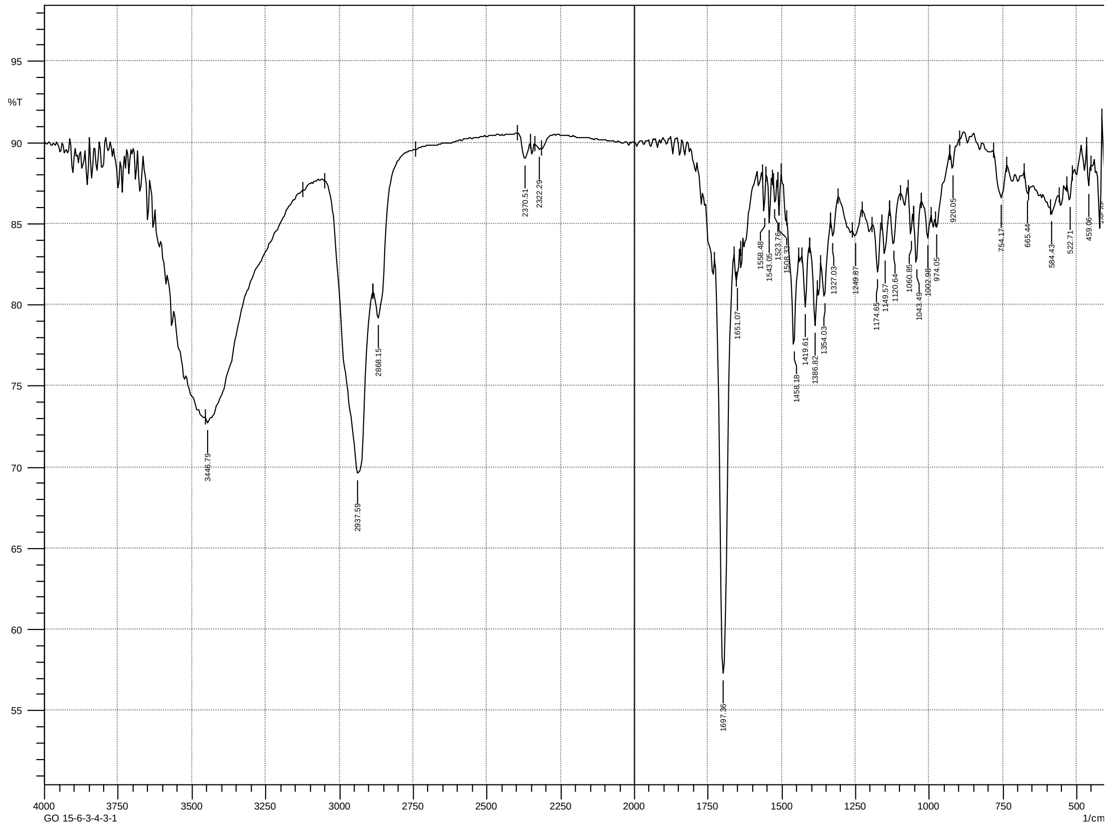

Figure S17. IR spectrum of **3**.

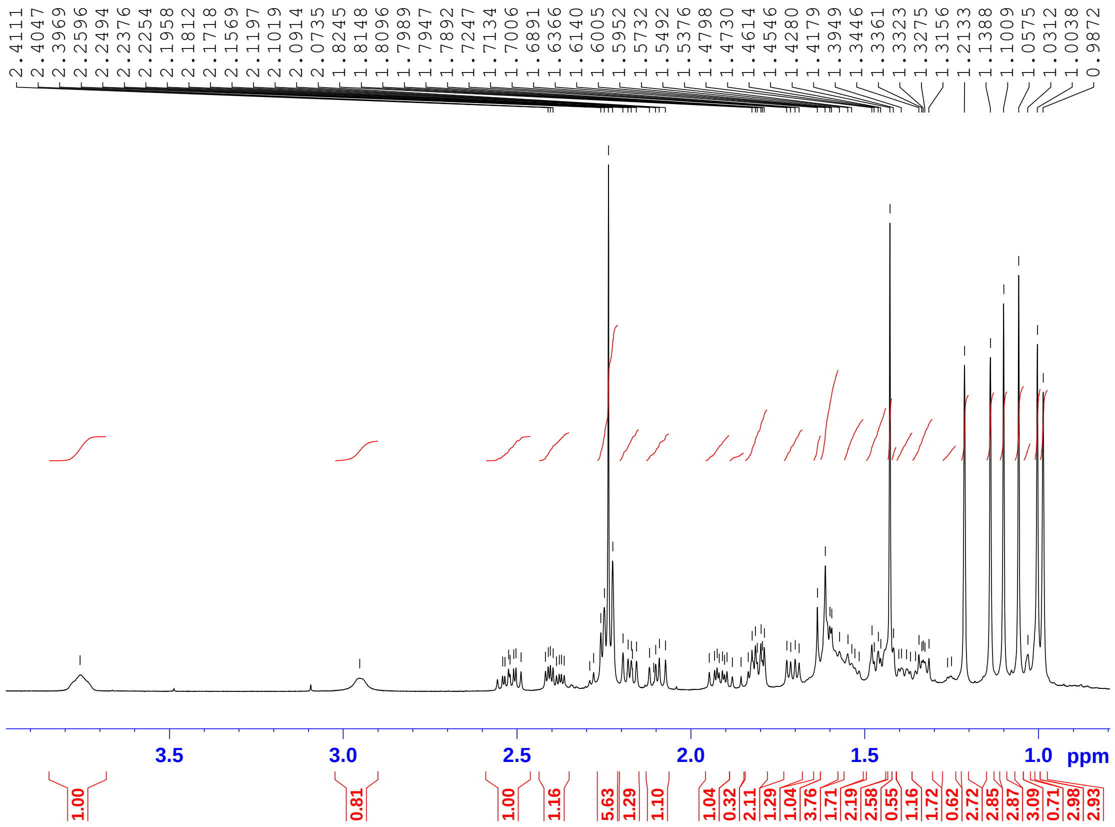

Figure S18. ^{1}H-NMR spectrum of **3** ($CDCl_3$, 500 MHz).

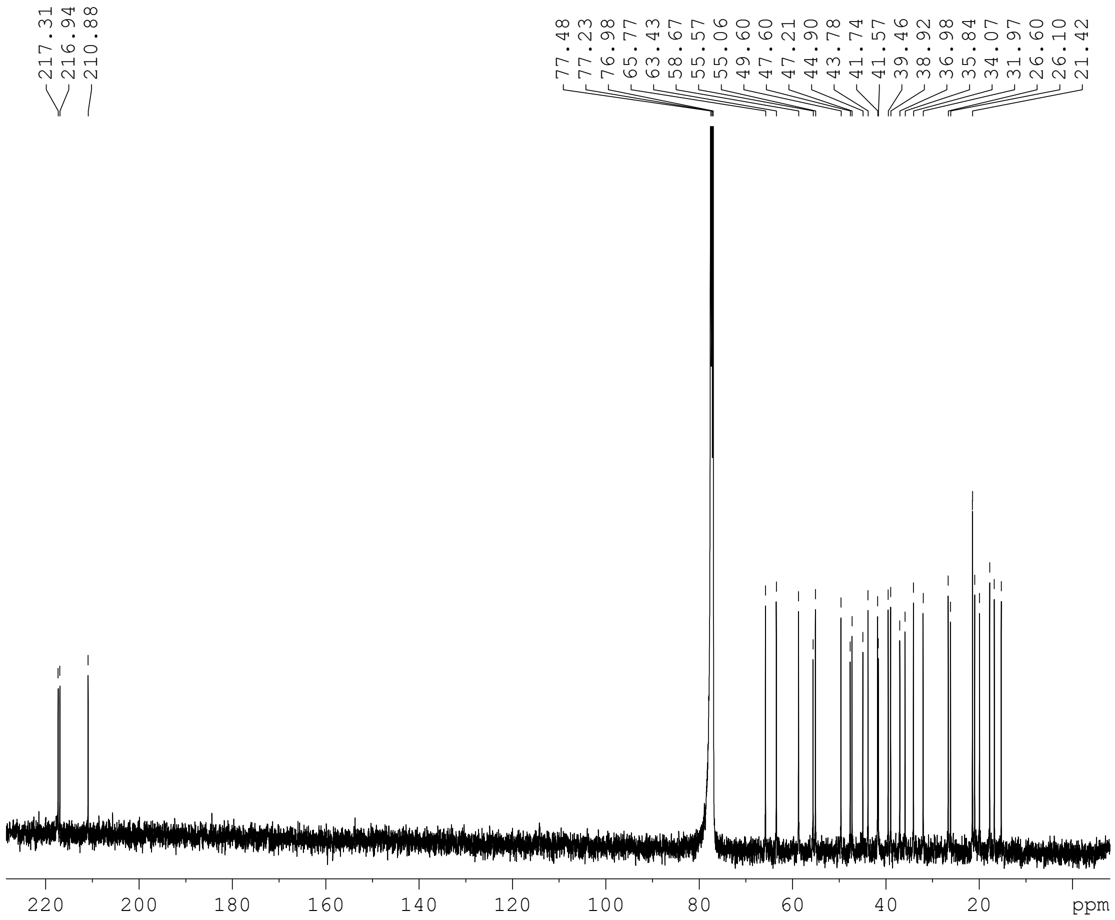

Figure S19. ^{13}C-NMR spectrum of **3** ($CDCl_3$, 125 MHz).

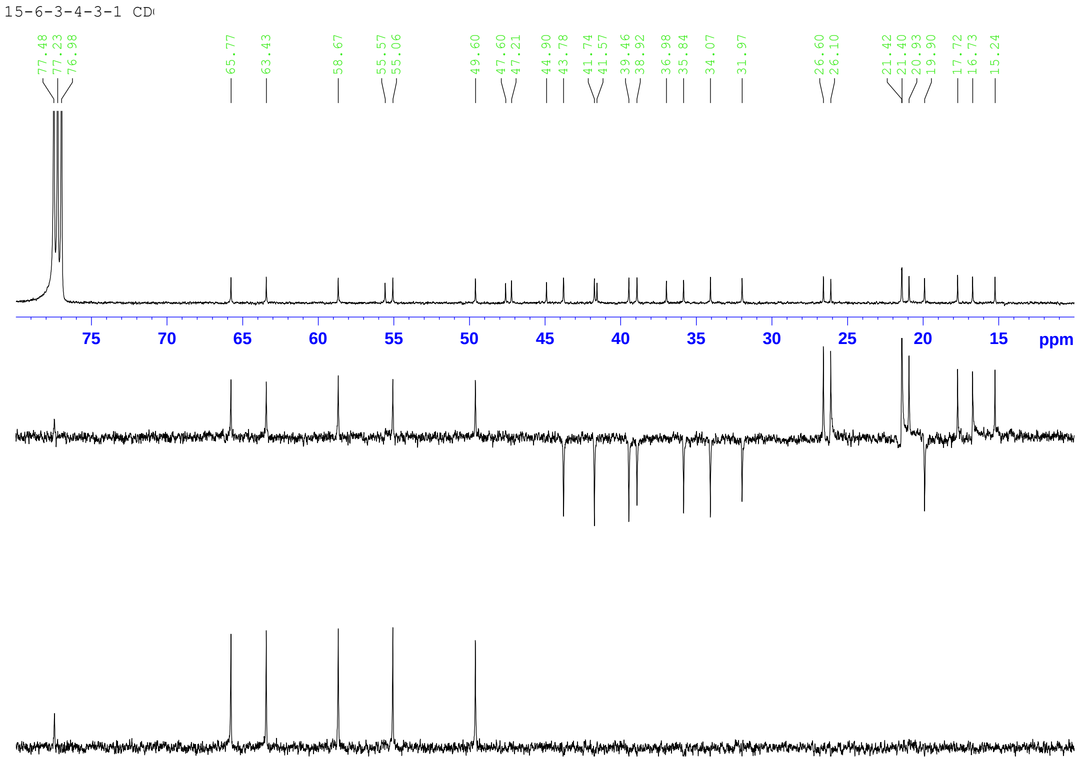

Figure S20. ^{13}C-DEPT spectrum of **3** ($CDCl_3$, 125 MHz).

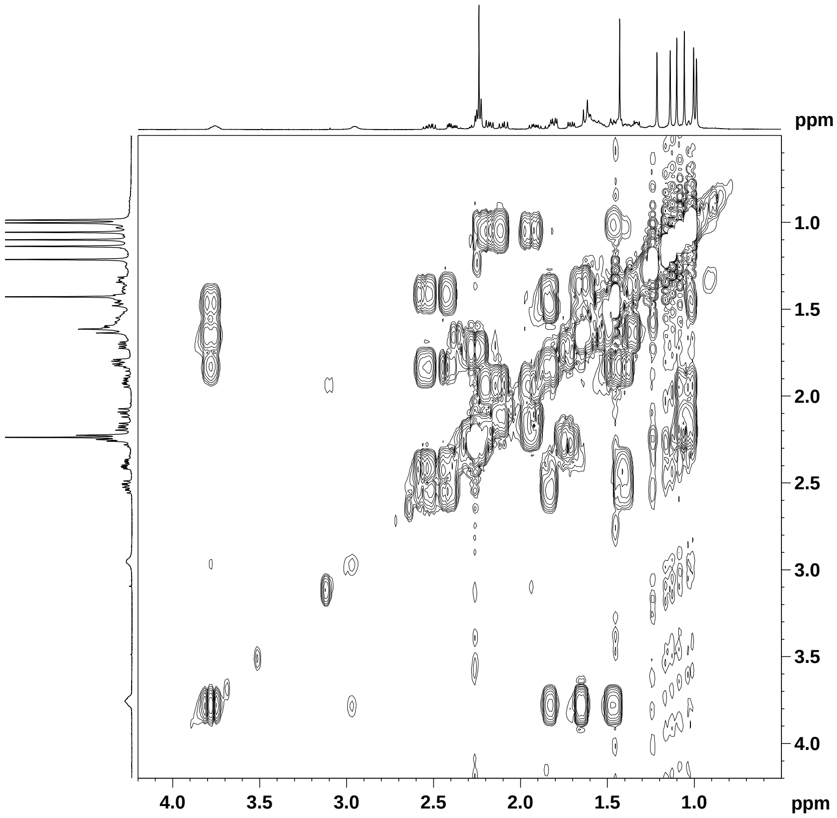

Figure S21. ^{1}H–^{1}H COSY spectrum of **3**.

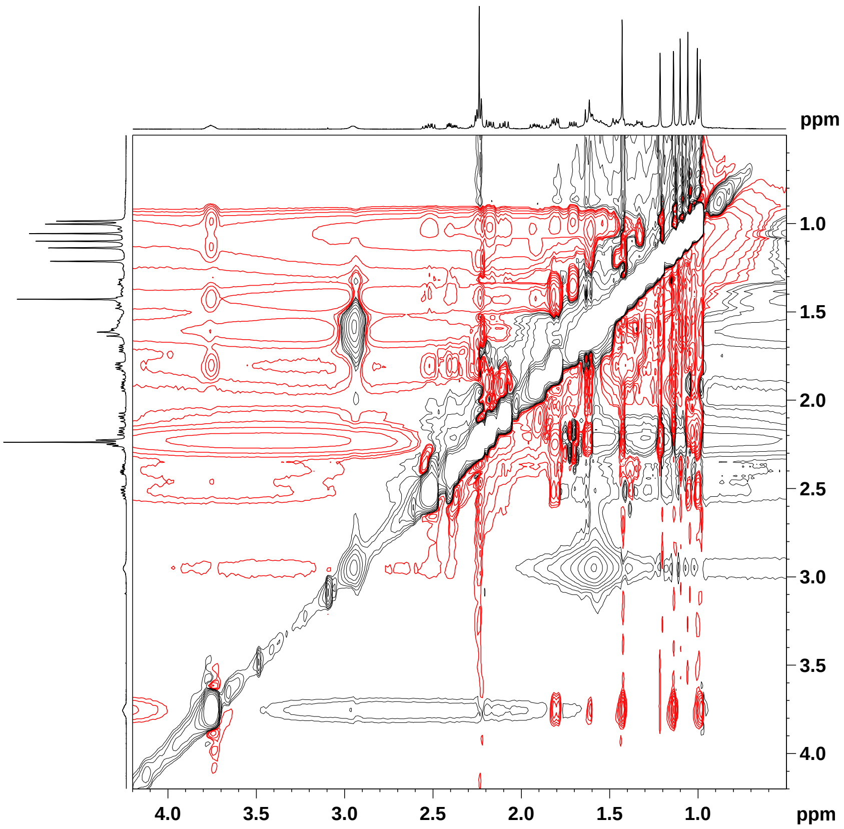

Figure S22. NOESY spectrum of **3**.

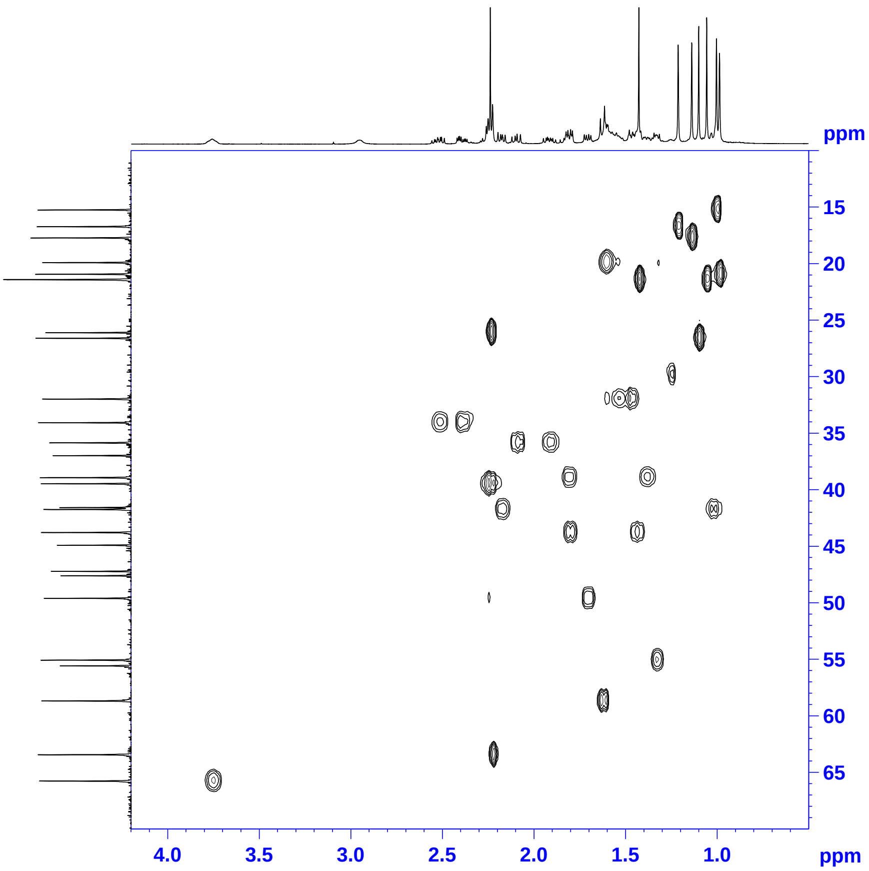

Figure S23. HSQC spectrum of **3**.

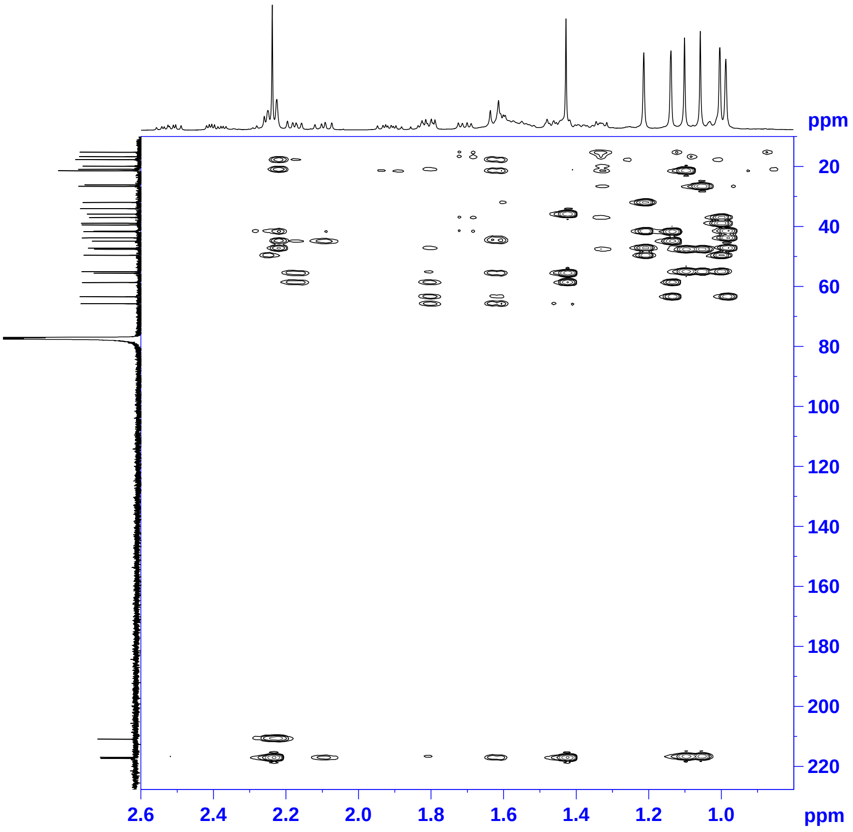

Figure S24. HMBC spectrum of **3**.
